# Disentangling natural and anthropogenic effects on benthic macroinvertebrate assemblages in western US streams

**DOI:** 10.1002/ecs2.4688

**Published:** 2023-11-09

**Authors:** C. Emi Fergus, J. Renée Brooks, Philip R. Kaufmann, Alan T. Herlihy, Ryan A. Hill, Richard M. Mitchell, Paul Ringold

**Affiliations:** 1 Oak Ridge Institute for Science and Education, U.S. Environmental Protection Agency, Corvallis, Oregon, USA; 2 US EPA, Office of Research and Development, Center for Public Health and Environmental Assessment, Pacific Ecological Systems Division, Corvallis, Oregon, USA; 3 Oregon State University, Department of Fisheries, Wildlife and Conservation Science, Corvallis, Oregon, USA; 4 US EPA, Office of Water, Washington, DC, USA

**Keywords:** multiscale drivers, regional analysis, riparian cover, stream biological condition, stream substrate, structural equation models

## Abstract

Stream macroinvertebrate assemblages are shaped by natural and human-related factors that operate through complex hierarchical pathways. Quantifying these relationships can provide additional insights into stream ecological assessment. We applied a structural equation modeling framework to evaluate hypothesized pathways by which watershed, riparian, and in-stream factors affect benthic macroinvertebrate condition in the Western Mountains (WMT) and Xeric (XER) ecoregions in the United States. We developed a conceptual model grounded in theory, empirical evidence, and expert opinion to evaluate the following hypotheses: (1) macroinvertebrate assemblages are primarily driven by proximal, in-stream factors (e.g., water quality and physical habitat); (2) anthropogenic land uses affect macroinvertebrates indirectly by altering in-stream characteristics; and (3) riparian vegetation cover attenuates land use effects. We tested our model separately on three measures of benthic macroinvertebrate assemblage condition: ratio of observed-to-expected taxonomic richness (O/E); a multimetric index (MMI); and richness of Ephemeroptera, Plecoptera, and Trichoptera taxa (EPT). In the WMT, site-level riparian cover, in-stream physical habitat (relative bed stability), and water chemistry (total nitrogen) were the top three predictors of macroinvertebrate assemblages, each having over two times the magnitude of effect on macroinvertebrates compared with watershed-level predictors. In the arid XER, annual precipitation and stream flow characteristics were top predictors of macroinvertebrate assemblages and had similar magnitudes of effect as in-stream water chemistry. Path analyses revealed that land use activities in the watershed and at the stream site degraded macroinvertebrate assemblages indirectly by altering relative bed stability, water quality, and riparian cover/complexity. Increased riparian cover was associated with greater macroinvertebrate condition by reducing land use impacts on stream flow, streambed substrate, and water quality, but the pathways differed among ecoregions. In the WMT, site-level riparian cover affected macroinvertebrate assemblages partly through indirect pathways associated with greater streambed stability and reduced total nitrogen concentrations. In contrast, in the XER, watershed-level riparian cover affected macroinvertebrate assemblages through greater specific stream power. Identifying the relative effects of and pathways by which natural and anthropogenic factors affect macroinvertebrates can serve as a framework for prioritizing management and conservation efforts.

## INTRODUCTION

Land use activities and water control structures (e.g., dams) impair the biological condition of streams through multiple pathways that can be variable and complex (Allan, 2004; Maloney & Weller, 2011; Riseng et al., 2011; Schmidt et al., 2018). Agriculture and urban development negatively affect stream physical structure and ecological functions by removing natural vegetation, promoting erosion, altering stream channel morphology and hydrologic regimes, and acting as sources of nutrients and other pollutants to streams. Dams alter fluvial-geomorphological processes and fragment stream networks and floodplain connectivity, which in turn harms aquatic biotic assemblages (Poff et al., 2007). Quantifying the relative effects of multiple anthropogenic disturbances and the varied pathways by which they operate is imperative to meet the goals and objectives of the US Clean Water Act and improve aquatic resource assessment and management. However, this is challenging because heterogeneity in natural hydrogeomorphic and climatic gradients interact to affect anthropogenic effects on streams (Allan, 2004; Birk et al., 2020; Grant et al., 2003; Riseng et al., 2011). Theory-based conceptual frameworks and appropriate analytic techniques can aid in separating the relative effects of natural and anthropogenic factors on stream condition.

Streams are commonly studied from a landscape ecology perspective, where hierarchically structured and spatially nested natural and anthropogenic features influence stream characteristics and aquatic assemblages (Fausch et al., 2002; Frissell et al., 1986; Thorp et al., 2006; Torgersen et al., 2022; Wiens, 2002). Within this framework, regional climate, geology, and topography are hypothesized to structure watershed-scale geomorphic, land cover, and hydrologic processes. In turn, these watershed characteristics influence stream hydromorphology, physical habitat conditions, and water chemistry concentrations, which determine potential aquatic community composition (Kaufmann & Hughes, 2006). Anthropogenic land use and dams can alter these relationships and lead to in-stream physiochemical alterations such as altered hydrologic regimes, degraded physical habitat, excess fine sediment, and poor water quality that adversely impact aquatic organisms (Heatherly et al., 2007; Helms et al., 2009; Hughes & Vadas, 2021; Meißner et al., 2019; Pitlick & Wilcock, 2001). From this landscape lens, watershed-scale natural and anthropogenic factors are hypothesized to indirectly affect stream biological condition, and several studies have supported this conceptualization with watershed-scale effects being mediated through proximal, in-stream physical and chemical conditions (Maloney & Weller, 2011; Riseng et al., 2011; Schmidt et al., 2018; Tang et al., 2020). Determining the relative importance of multiscaled natural and anthropogenic features and the mechanistic pathways by which they affect stream biota can further our understanding of stream ecosystems in the landscape and better inform management actions.

Protecting and/or creating riparian buffers are considered cost-effective management options to support stream ecosystems and mitigate the effects of watershed-scale land use activities (Cole et al., 2020; Hughes & Vadas, 2021). Natural riparian vegetation cover provides direct benefits to stream ecosystems by stabilizing streambanks, shading nearshore areas (which can moderate stream temperature and reduce light for primary production), and supplying organic matter and woody debris to streams that support secondary production and provide substrate for macroinvertebrates (Feld et al., 2018; Gregory et al., 1991; Palt et al., 2022; Turunen et al., 2021). But the effectiveness of riparian buffers in mitigating human disturbances by slowing runoff and reducing or preventing sediment, nutrients, and other pollutants from entering streams is not consistent across studies (Hughes & Vadas, 2021; Le Gall et al., 2022; Wahl et al., 2013). These relationships can be affected by the extent and intensity of land use in the watershed, which limit the efficacy of riparian buffers as management tools (Feld et al., 2018; Turunen et al., 2019). Quantifying the relative effects of riparian vegetation cover requires analytic approaches that can model the complex pathways by which anthropogenic disturbances, riparian features, and in-stream stressors cascade down to affect stream biological assemblages (Leitão et al., 2018).

Structural equation modeling (SEM) offers an analytic framework to evaluate hypothesized networks of interrelated variables (Grace et al., 2010) and has been applied to address complex questions pertaining to coupled natural and anthropogenic systems (e.g., Fergus et al., 2022; Meißner et al., 2019; Palt et al., 2022; Schmidt et al., 2018; Tang et al., 2020). Unlike conventional statistical analyses like multiple regression, SEM offers greater application and flexibility to model complex systems by mathematically representing multivariate networks of response and predictor variables. Within this framework, systems-level hypotheses about causal pathways can be evaluated and the effects of multiple drivers can be disentangled, allowing estimation of their direct and indirect effects through mediating variables. This modeling approach is well suited to evaluate the multiscale factors hypothesized to structure stream ecosystems.

**FIGURE 1:**
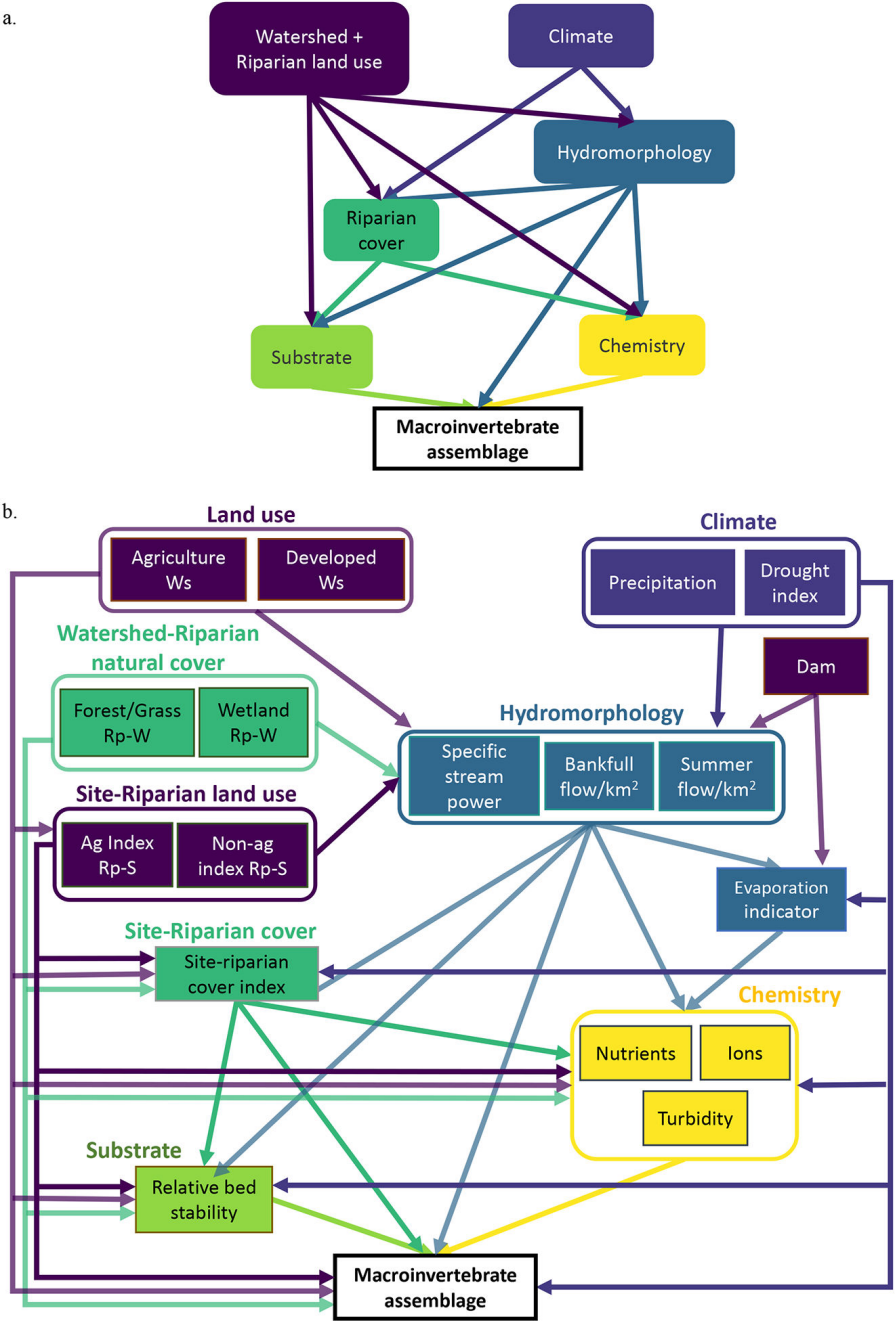
Hypothesized predictors of macroinvertebrate assemblages in streams in the Western United States. (a) Metamodel of conceptual drivers and generalized relationships affecting macroinvertebrate assemblages. (b) Path analysis model with predictor variables from National Rivers and Streams Assessment and StreamCat. Variables are hierarchically structured and grouped into driver categories that are color-coded: land use (watershed and riparian), climate, riparian natural cover, hydromorphology, substrate, and chemistry. Land use activities are quantified at two spatial scales: the watershed (Ws) and in the riparian area at the stream site (Rp-S). Riparian natural cover is quantified at two spatial scales: the riparian buffer within the watershed (Rp-W) and in the riparian area immediate to the stream site (site-riparian). See Table 1 for variable descriptions.

In this study, we evaluated hypothesized pathways by which hierarchically structured natural and anthropogenic factors affect macroinvertebrate condition in western US streams using an SEM framework. We developed a conceptual model that was based on theory, empirical evidence, and expert opinion (Figure 1) and evaluated the following hypotheses: (1) proximal, in-stream variables are primary drivers of benthic macroinvertebrate assemblages, (2) anthropogenic disturbances at watershed and riparian scales affect macroinvertebrate assemblages indirectly by altering in-stream conditions, and (3) riparian vegetative cover attenuates land use effects on macroinvertebrate assemblages. We converted the conceptual model to a path analysis model (a structural equation model composed of observed variables) to test our hypotheses using variables from the US EPA National Rivers and Streams Assessment (NRSA) and StreamCat datasets. We evaluated the generality and robustness of our hypothesized model by applying a path analysis model to predict three benthic macroinvertebrate indices that are commonly used to characterize stream biological condition: observed-to-expected taxa presence ratios (O/E), a multimetric index (MMI), and richness of sensitive taxa groups Ephemeroptera, Plecoptera, and Trichoptera (EPT). We examined ecoregional differences in drivers and relationships by applying our models to two western ecoregions: Western Mountains (WMT) and Xeric (XER) (Herlihy et al., 2008; Kaufmann, Hughes, Paulsen, Peck, Seeliger, Weber, et al., 2022). With these models, we can gain a deeper understanding of the pathways by which natural and anthropogenic factors affect stream ecosystems in the Western United States.

## METHODS

### Overview of approach

We applied an SEM framework to evaluate our hypothesized model of natural and anthropogenic drivers of stream macroinvertebrate assemblages. We first developed a metamodel conceptualizing broad classes of drivers of stream macroinvertebrate condition (Figure 1a) based on theory, empirical evidence, and insights from experts (Appendix S1: Table S1). We adapted this metamodel into a path analysis model (Figure 1b) that depicted hypothesized directional relationships among specific variables in the datasets (Table 1) that we color-coded by driver categories. We applied the path analysis model separately to predict three different macroinvertebrate response variables within two ecoregions for a total of six models. With the path analysis models, we could evaluate the validity of our hypothesized network of multiscale drivers of stream ecosystem condition and quantify their relative effects on macroinvertebrate assemblages.

### Datasets and study setting

We used data collected by NRSA over three survey periods: 2008–2009, 2013–2014, and 2018–2019. NRSA is designed to make inference on the condition of the greater population of streams and rivers in the conterminous United States (CONUS) using a stratified random sample design and collecting in situ information on a suite of variables to characterize stream water quality, in-stream and riparian habitat, and biological condition at regional and national extents (USEPA, 2013). NRSA sites were selected from the National Hydrography Dataset (NHD) Plus (McKay et al., 2012) based on a probability-based, randomized sampling design (USEPA, 2020a) with a target population of all streams and rivers in CONUS with flowing water during the sampling period (April–September). CONUS was delineated into nine ecoregions based on aggregating Omernik Level-III ecoregions (Herlihy et al., 2008). Each of the NRSA surveys sampled over 2000 stream and river sites, with about 40% of sites resampled in later surveys to assess year-to-year variation, and about 10% of sites within a survey period randomly selected for a second visit during the year to assess within-year variation. More details of the NRSA sampling design can be found in Olsen and Peck (2008) and the USEPA technical report (2020a).

Our analyses were restricted to wadeable streams in the WMT and XER ecoregions. NRSA defines wadeable sites as having continuous water flow and >50% of the sample reach being wadeable (USEPA, 2012). NRSA field sampling and lab processing protocols are provided in US EPA manuals (2013, 2020a) and Hughes and Peck (2008) and are described briefly here. NRSA sample sites were defined as stream reaches 40 times as long as their mean wetted widths, with a minimum of 150 m to a maximum reach of 4000 m. Water samples were collected from the middle of the channel and shipped to a central laboratory to be analyzed for a suite of chemical constituents. Water subsamples were analyzed for water stable isotope ratios (δ^2^H, δ^18^O) performed by the Integrated Stable Isotope Research Facility at the Pacific Ecological Systems Division of the US EPA and were used to estimate an index of evaporation in the stream. Benthic macroinvertebrate samples and physical habitat and wadeable stream measurements were taken along 11 evenly spaced transects along the sample reach. Benthic macroinvertebrate samples were collected using a 0.5-m D-frame kick-net (500-μm mesh) at alternating left, mid, and right positions of the streambed at the transects. Samples were composited and preserved with ethanol. In the laboratory, organisms were identified to the lowest practical taxonomic level, typically genus, using standard keys and references. Physical habitat data were collected from longitudinal profiles and from transects and channel/riparian plots evenly distributed along the site reach (Kaufmann et al., 1999; Kaufmann, Hughes, Paulsen, Peck, Seeliger, Weber, et al., 2022). Riparian vegetation, anthropogenic disturbances, channel incision, stream bed particle size, and wetted and bank-full channel dimensions were recorded at transects and in-stream plots.

### Data processing

**FIGURE 2:**
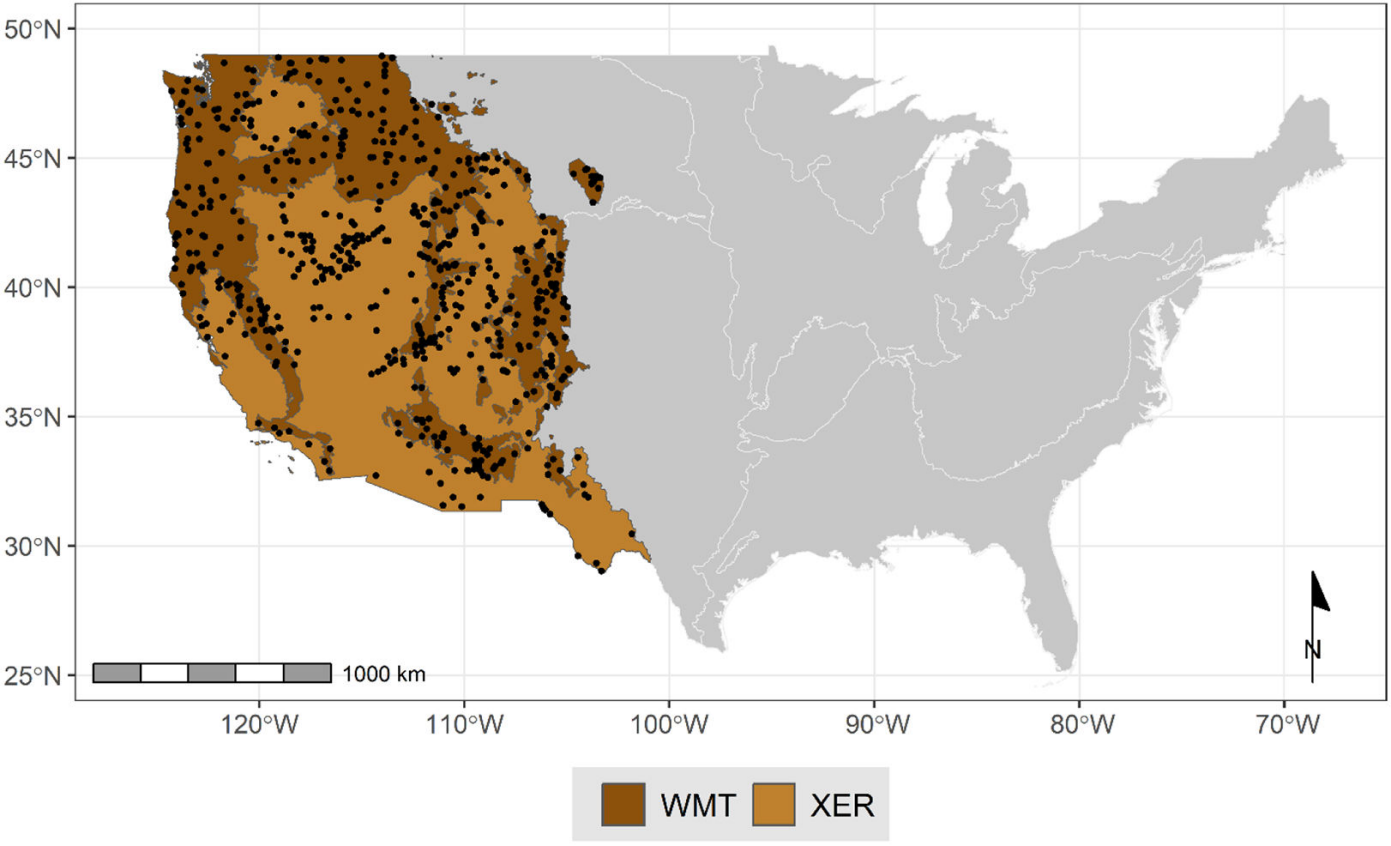
Stream reach sites from the National Rivers and Streams Assessment in the Western Mountains (WMT) and Xeric (XER) ecoregions in the conterminous United States. WMT = 323 sites and XER = 272 sites.

We compiled data from three NRSA survey periods to create a dataset for the path analysis models. In the processed dataset, we retained all observations from the 2008–2009 survey and new stream sites added in subsequent survey periods. If the site was visited more than once during the sample period, we used only the first visit within a sample period to create a dataset of unique stream sites across survey periods without repeat observations. The final processed datasets included single visits from 323 wadeable stream sites in the WMT and 272 sites in the XER ecoregions (Figure 2).

### Derived NRSA variables

Variables characterizing stream macroinvertebrate assemblages, hydromorphology, riparian condition, and physical habitat were derived from NRSA field-collected data.

#### Macroinvertebrate assemblages

Three variables characterizing the condition of stream macroinvertebrate assemblages were derived from NRSA macroinvertebrate taxon data: O/E, a benthic macroinvertebrate MMI, and richness of sensitive EPT taxa. The variables reflect different aspects of macroinvertebrate assemblages that may respond differently to environmental characteristics and stressors.

O/E richness is calculated as the ratio of the number of observed taxa (*O*) to the number of expected taxa (*E*) under least-disturbed (reference) conditions (Hawkins et al., 2000). O/E values can be interpreted as a loss of taxa where an O/E value of 0.40 indicates that 40% of the expected taxa at a site were observed and 60% of taxa were lost. O/E scores for the Western United States were generated from steps outlined in USEPA (2020a). *E* was predicted from regional least-disturbed reference sites (Stoddard et al., 2006) in the west ecoregion (WMT + XER) using random forest with the following predictors: watershed area, mean annual and mean maximum temperature, mean precipitation accumulation, predicted stream temperature, and mean elevation. A stream site in reference conditions would ideally have an O/E = 1, but values can vary among reference sites and are affected by modeling error and sampling variation. In the west ecoregion, reference wadeable sites had a mean O/E of 0.99 with a SD of 0.23. Individual taxa O/E values are most reliably interpreted relative to the entire O/E assemblage distribution in reference sites.

*A MMI* combines multiple community assemblage metrics based on life history characteristics and tolerance to environmental variables (e.g., functional feeding group, tolerance to disturbance) into a single numerical index to assess macroinvertebrate assemblage condition. Macroinvertebrate MMIs in NRSA were developed separately for each of the nine aggregated ecoregions based on the methods from the Wadeable Stream Assessment survey (Stoddard et al., 2008) and are described in USEPA (2020a). Candidate community metrics were evaluated for the ecoregional MMIs and classified into six categories reflecting important community structure characteristics: (1) richness, (2) evenness/diversity, (3) taxonomic composition, (4) functional feeding groups, (5) habit (predominant behavior), and (6) tolerance of environmental stressors. Metrics were assessed based on their range, reproducibility, ability to calibrate for natural variation, responsiveness to anthropogenic disturbance, and metric redundancy. One metric per category was selected and scored for the composite MMI variable. MMI values range from 0 to 100, with higher scores indicating better biological condition. The selected metrics used to derive MMIs in the WMT and XER ecoregions are listed in Appendix S1: Table S2. Mean and (SD) values of MMI in WMT and XER wadeable reference sites were 53 (15.6) and 56 (17.9), respectively.

*EPT* orders are composed of pollution-sensitive insect species with relatively specific habitat requirements, and thereby can be informative integrative metrics of stream biotic condition (Gieswein et al., 2017). EPT taxa richness, calculated as the total number of unique EPT taxa at a site, is commonly used in biomonitoring programs and cited as one of the most useful macroinvertebrate metrics by US state agencies to assess streams and address state and federal environmental requirements (Carter & Resh, 2013). The advantages of EPT taxa richness compared with other macroinvertebrate variables are that it is relatively easy to calculate, and values can be compared across bioassessment programs in contrast to O/E and MMI, which may be derived using different approaches across programs and rely on designating reference sites.

#### Hydromorphology

Stream hydromorphology variables in the model include specific stream power, bank-full flow (discharge/area), summer flow, and an indicator of evaporation. Specific stream power index was calculated by multiplying the slope of the stream reach by thalweg depth and dividing by the wetted width. This metric also serves as a coarse index of bed shear stress in the stream. Bank-full and summer flow were calculated from field measurements of physical habitat structure using the Chezy formulation as adapted by Kaufmann et al. (2008). We derived an indicator of evaporation of stream water using water stable isotopes (δ^2^H, δ^18^O). We calculated deuterium excess (d-excess, Equation 1), an index of how much evaporation has affected the isotopic value of the water sample (Brooks et al., 2014; Clark & Fritz, 1997), and then took the inverse of d-excess to scale the values for easier interpretation (Equation 2).


(1) $$d\text{-excess}=\delta^{2}H-8\delta^{18}O.$$

(2) Evaporation indicator=(d-excess−10)×−1.

Higher values of the evaporation indicator signify greater evaporation of surface waters flowing to the site.

#### Riparian cover and land use

An index of riparian vegetation cover and structural complexity (site-riparian cover index) at the stream site was calculated from NRSA visual estimates of woody vegetation cover in the ground layer (<0.5 m), mid-layer (0.5–5.0 m), and canopy layer (>5.0 m). This index conveys both abundance and structural complexity of riparian vegetation with a theoretical maximum value of 3.0 if there is 100% woody cover in each of the three vegetation layers. Riparian anthropogenic disturbances were recorded as the presence and proximity of streamside human activities in each riparian plot (Kaufmann et al., 1999). Proximity-weighted disturbance indices were calculated by summing the number of riparian stations where particular land use activities were observed, weighting each observation according to its proximity to the stream, and then averaging over all transects. Riparian disturbance categorized as agriculture (Ag index Rp-S) included row crops, pastures, and hayfields. Riparian disturbance categorized as nonagricultural (Non-ag index Rp-S) included buildings, dikes and bank revetment, landfill/trash, logging, mining, developed parks or lawns, pavement or cleared lots, pipes, and roads.

#### Substrate

Relative bed stability was calculated as the ratio of the geometric mean surface particle diameter to the maximum mobile diameter at bank-full flow (Kaufmann et al., 1999, 2008; Kaufmann, Hughes, Paulsen, Peck, Seeliger, Weber, et al., 2022). Mean bed particle size was estimated based on systematic streambed particle sampling. The maximum mobile particle diameter at bank-full flow was based on estimated streambed shear stress calculated from stream site channel slope, channel dimensions, and hydraulic roughness estimated for bank-full flow conditions. Relative bed stability values less than 1 are indicative of mobile, unstable streambeds with potentially excess fine sediment. Values greater than 1 indicate stable streambeds, usually consisting of coarse particles or bedrock.

### Geospatial watershed variables

We used landscape and climate data from StreamCat, a geospatial database that compiles multithematic and multiscaled landscape metrics for millions of US streams and rivers (Hill et al., 2016). We joined StreamCat data to NRSA sites based on the unique stream ID. Additional geospatial variables that were not available in StreamCat at the time of analysis were obtained from their source geographic layers (Table 1) and summarized following data processing steps used in StreamCat. Land use and cover variables in StreamCat originally come from the USGS National Land Cover Database (NLCD). We joined NLCD metrics with NRSA observations by year to match land cover/use with stream observations at roughly the same time period, for example, joining NLCD 2008 variables with NRSA 2008–2009.

#### Watershed land use

We characterized agriculture and urban development in the watershed by aggregating NLCD land use classes (Agriculture Ws = [% Row crop + % Hay] and Developed Ws = [% Open space + % Low + % Medium + % High-intensity development]). Dam storage, originally from the National Anthropogenic Barrier Dataset (Ostroff et al., 2013), estimates the volume of all reservoirs per unit area in the upstream watershed. We divided dam storage capacity by watershed runoff (McCabe & Wolock, 2011) to scale to watershed size.

#### Watershed riparian cover

Riparian vegetation cover in the watershed was estimated by aggregating NLCD land cover classes within a 100-m buffer along the NHD streamline. Riparian cover classes included Forest/Grass Rp-W (Deciduous + Coniferous + Mixed forest + Shrub + Grassland) and Wetland Rp-W (Woody + Herbaceous Wetland).

#### Climate

Climate variables in our model included annual precipitation (PRISM Climate Group) and an index of drought based on Palmer Hydrologic Drought Index (PHDI) (Vose et al., 2014). Annual precipitation was calculated as the total precipitation during the year preceding when NRSA sites were sampled. PHDI values indicate the severity of a wet (positive) or dry (negative) period and account for longer term dryness related to local precipitation, temperature, and available water capacity of the soil that may affect water storage, streamflow, and groundwater levels. We calculated a drought index from PHDI by multiplying mean annual PHDI during the respective survey calendar year by −1 so that positive values indicate dry conditions and negative values indicate wet conditions.

### Conceptual model of drivers of stream macroinvertebrate assemblages

We developed a metamodel depicting relationships among hypothesized natural and anthropogenic drivers of stream macroinvertebrate assemblage condition in western US streams (Figure 1a). The metamodel is a conceptualization of theoretical constructs (e.g., general drivers) and causal processes (e.g., mechanisms) of the system of interest that is rooted in theory, empirical evidence, and expert opinion (Grace et al., 2010). We hypothesized that benthic macroinvertebrate assemblages are affected by multiscaled natural and anthropogenic drivers that we classified into the following categories: watershed and riparian land use, climate, riparian natural cover, stream hydromorphology, substrate, and water chemistry. We expected that proximal features related to physical habitat and water chemistry are direct and dominant drivers of benthic macroinvertebrate assemblages. We hypothesized that anthropogenic disturbances have the potential to affect multiple dimensions of stream ecosystems and thereby indirectly alter macroinvertebrate assemblages by directly impairing stream channel morphology, hydrology, riparian cover and complexity, in-stream physical habitat, and water quality. Climate was expected to directly affect hydrology and riparian cover/complexity, which in turn affect stream physical habitat and water chemistry.

Using variables in NRSA (variables measured at the site) and StreamCat datasets (GIS-derived variables), we converted the conceptual metamodel of hypothesized drivers of stream macroinvertebrates into a path analysis model (Figure 1b, Table 1; Appendix S1: Table S1). The path analysis model did not include direct measures of channel alteration, which can range from removal of woody debris to reconfiguration of river channels (Elosegi & Sabater, 2013), because that detailed level of information was not systematically collected on the NRSA sites. Rather, we incorporated potential causes and effects of channel alteration in the model by including pathways linking stream hydromorphology variables and site-level riparian land use indices to in-stream habitat, water chemistry, and macroinvertebrate assemblage. We included covariances among the stream hydromorphology variables (specific stream power, summer flow, and bank-full flow) and among water chemistry variables (e.g., total nitrogen [TN] and sulfate) in the model because we expected these variables to be driven by similar factors and to be correlated with one another. With the path analysis model, we can test whether these relationships are supported by the data and evaluate if our representation of the system is incomplete. Variables were colored by driver categories (land use, climate, hydromorphology, riparian natural cover, substrate, and chemistry).

### Analytical steps

All data processing and analyses were performed using R Statistical Software (Team, 2022). Mixed-effects models to estimate metric precision (signal-to-noise) were performed using the lme4 package (Bates et al., 2015). Path analysis models were performed using the lavaan package (Rosseel, 2012). We evaluated univariate and multivariate normality of variables in the model by inspecting distribution plots and using Shapiro–Wilk (univariate) and Mardia’s (multivariate) normality tests. Stream hydromorphology, site-riparian cover index, relative bed stability, and water chemistry variables were log_10_ transformed and percent watershed variables were arcsine square root transformed.

#### NRSA signal: noise and maximum $R^{2}$ estimates

We evaluated the precision of the NRSA macroinvertebrate assemblage, physical habitat, hydrological, and water chemistry variables in the ecoregional datasets by calculating signal-to-noise ratios (S:N). Variables with S:N < 2.0 indicate that the variance among sites (signal) is less than two times the variance within a site (noise) (Kaufmann et al., 1999). This low precision can limit the strength of association between response and predictor variables and set an upper limit on the maximum possible $R^{2}$ value in regression and path analyses (Kaufmann et al., 1999; Stoddard et al., 2008). We calculated S:N and maximum $R^{2}$ for NRSA response variables using the full ecoregional datasets with repeat visits within a sampling period. Signal-to-noise ratio was estimated from a null mixed-effects model with the stream site ID and year as random effects. Treating the site ID and year as a random effect partitioned the within-site temporal (noise) variance component from the remaining variation (signal) component. Maximum $R^{2}$ was derived from S:N:

(3) $$\max\;R^{2}=\frac{\text{S:N}}{\left(1+\text{S:N}\right)}.$$

Quantifying the precision of NRSA variables will let one set reasonable expectations to evaluate the path analysis model performance given underlying measurement variance in the variables.

#### Path analysis model

We evaluated the generality and robustness of our hypothesized path analysis model by applying it to three separate benthic macroinvertebrate indices (O/E, MMI, and EPT) in two ecoregions in the Western United States (WMT and XER) for a total of six models. The path analysis models were initially fit using robust maximum likelihood estimation methods. We iteratively trimmed nonsignificant paths (*p* < 0.05) from the model one at a time and removed variables that did not have a direct or indirect path to the benthic response variable. We referenced modification indices (values indicating the expected change in chi-square goodness of fit when path coefficients were added to the model) and ecological theory to determine whether additional pathways, namely correlations, would improve the model and were consistent with our a priori hypotheses. The overall model fit was evaluated using several parameters and thresholds: comparative fit index (CFI > 0.90), Tucker–Lewis index (TLI > 0.90), root mean square error of approximation (RMSEA < 0.08), and standardized root mean square residual (SRMR < 0.08). The chi-square global test is used to evaluate whether the model-implied variance structure is significantly different from the observed structure. This test is commonly used to evaluate SEMs but is sensitive to sample size, where analyses of larger sample sizes (>200) tend to detect small discrepancies between the modeled covariance matrix and observed covariance (Peugh & Feldon, 2020). Therefore, we evaluated the performance of the models using the other fit statistics highlighted above. Once a model met the fit criteria, we estimated path coefficients using Bollen–Stine bootstrapping (*n* = 1000) to accommodate non-normal distributions (Bollen & Stine, 1992). We reexamined path coefficient estimates and removed paths with *p* values >0.05 one at a time and repeated the above steps in an iterative process until the model had only significant paths.

We assessed the relative importance of predictors on each of the three stream macroinvertebrate response variables by calculating their total effect and comparing across driver categories. The total effect of a predictor is calculated as the sum of the standardized direct and indirect effects on a response, where the direct effect is the path coefficient linking the predictor directly to the response and the indirect effect is calculated as the product of sequential path coefficients linking the predictor to the response through other variables. The total effect is a more comprehensive assessment of a predictor’s effect on macroinvertebrate assemblages that take into account the multiple pathways by which they could operate.

## RESULTS

### NRSA signal:noise and maximum $R^{2}$ estimates

Signal:noise ratios for the NRSA response variables in the WMT and XER datasets ranged from low precision (<2.0) to high precision (>9.0) (Table 2). Macroinvertebrate O/E in the WMT had an S:N of 1.74, indicating that variance in O/E among sites in the WMT was less than twice as high as variance within the same site. Subsequently, the maximum theoretical $R^{2}$ for O/E in the WMT was estimated to be only 0.64. In contrast, MMI and EPT in the WMT had moderate precision with S:N of 4.18 and 3.73, respectively, and maximum $R^{2}$ values of 0.81 and 0.79. All three macroinvertebrate assemblage variables in the XER ecoregion exhibited moderate precision with S:N ranging from 2.47 for MMI to 4.05 for EPT. Most of the environmental response variables in both ecoregions had moderate to high precision and maximum theoretical $R^{2}$ close to 1. The exceptions being relative bed stability in the XER ecoregion, which had lower precision (S:N = 3.70) compared with relative bed stability in the WMT (S:N = 12.85) and evaporation index, which had an S:N ~ 2 in both the WMT and XER. The low S:N for evaporation index can be attributed to high variance of evaporation index values within a site across sampling periods that are likely due to drought and wet periods during the NRSA surveys.

### Path analysis model results

**FIGURE 3:**
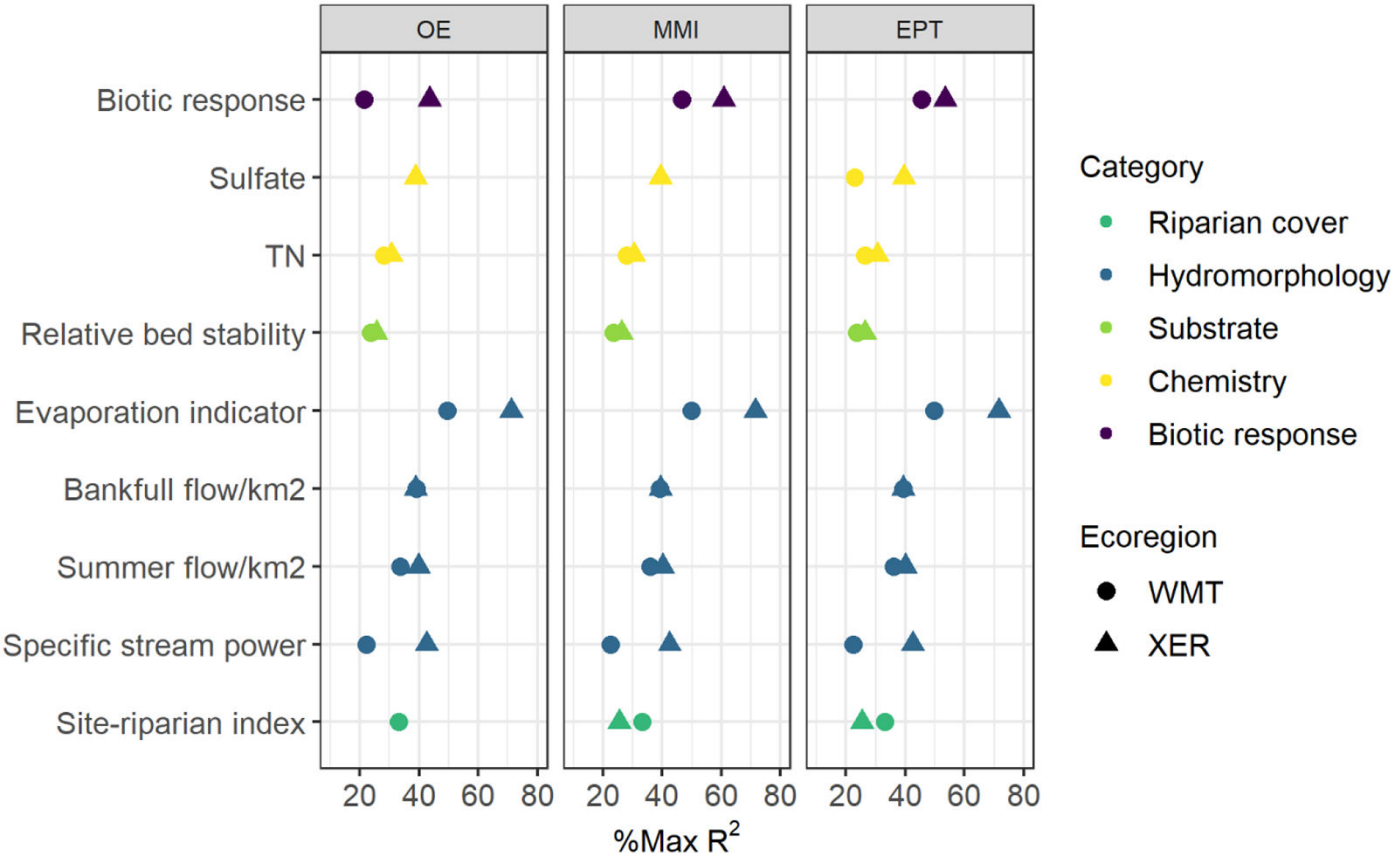
Percentages of the maximum $R^{2}$ of response variables explained by the path analysis models. $R^{2}$ of response variables was scaled by the theoretical maximum $R^{2}$ that could be achieved given measurement error to evaluate model performance. EPT, Ephemeroptera, Plecoptera, Trichoptera taxa richness; MMI, multimetric index; O/E, observed:expected; TN, total nitrogen; WMT, Western Mountains; XER, Xeric.

The final path analysis models were deemed to have good-to-acceptable model fit (Table 3), indicating that our conceptual model was supported by the data. After removing nonsignificant paths and variables not connected to the macroinvertebrate responses, the final models had CFI and TLI values >0.90 and RMSEA and SRMR <0.08. In the WMT, the final path analysis models accounted for over a third of the variation in benthic MMI ($R^{2}=0.38$) and EPT (0.36) but only 14% of the variation in O/E (Table 4). Similarly, in the XER ecoregion, the models accounted for more variation in MMI ($R^{2}=0.43$) and EPT (0.43) than O/E (0.32). The final models predicted other stream attributes (e.g., flow, substrate, water chemistry) relatively well with $R^{2}$ ranging from 0.20 to 0.48. Scaling the model $R^{2}$ by the maximum theoretical $R^{2}$ allowed for evaluating the model performance after accounting for precision (measurement variance) of the response variable. After scaling $R^{2}$ values, the path analysis models accounted for at least 20% or more of the maximum possible $R^{2}$ among the predictor variables (Figure 3). For example, after adjusting for the low precision of O/E in the WMT, the model accounted for 21.5% of O/E variance compared with only 14% from the unadjusted $R^{2}$. Response variables with moderate to high precision were less influenced by the problems associated with low precision and poor regression performance.

#### Proximate drivers of macroinvertebrates

**FIGURE 4:**
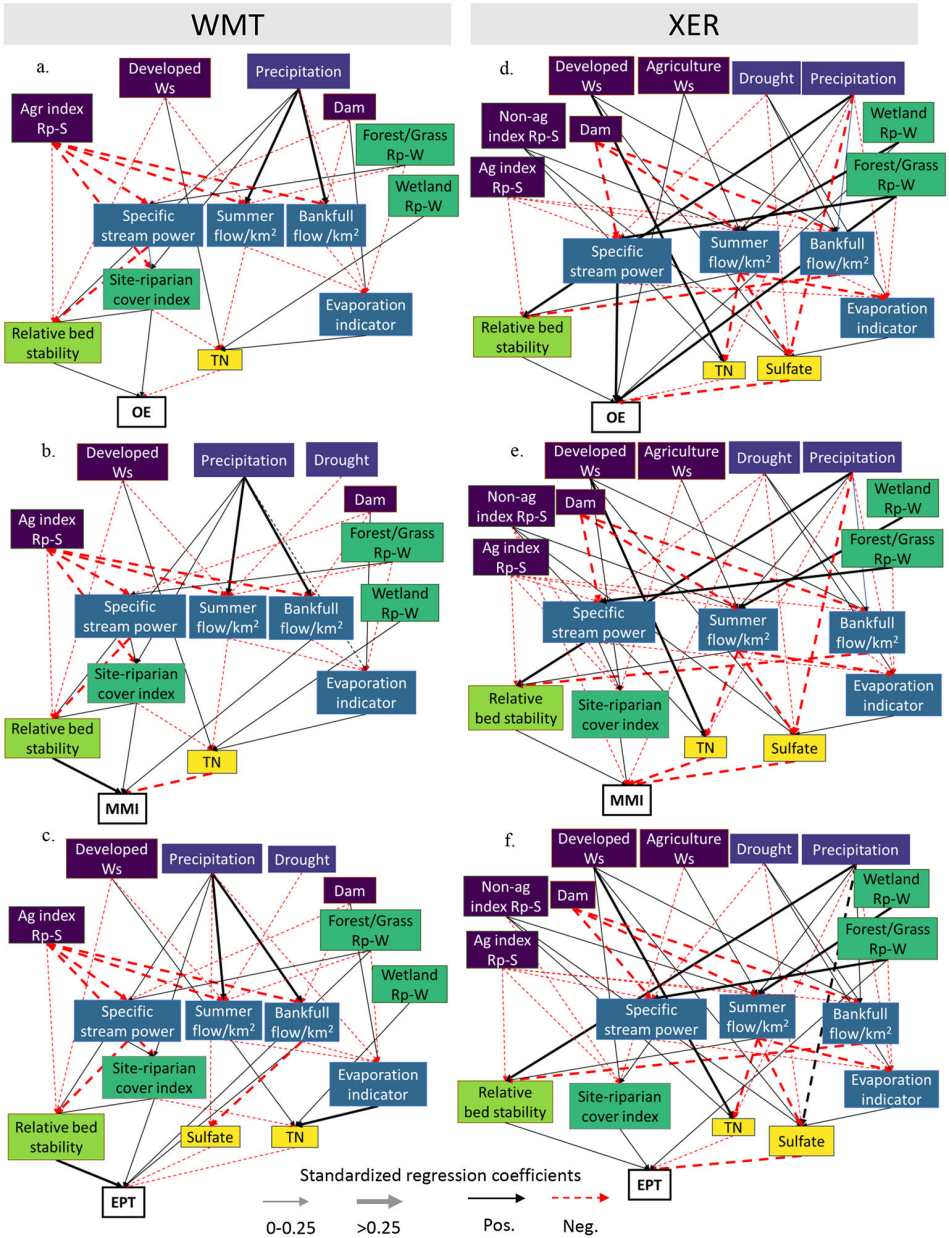
Macroinvertebrate path analysis diagrams by ecoregion. Path analysis diagrams for macroinvertebrate observed:expected (O/E), multimetric index (MMI), and Ephemeroptera, Plecoptera, Trichoptera taxa richness (EPT) are presented for the Western Mountains (WMT) (a–c) and Xeric (XER) (d–f) ecoregions. Single-headed arrows represent standardized path coefficients of predictors on response variables (i.e., direct effects). Solid black arrows indicate positive relationships, dashed red arrows indicate negative relationships, and the width of the arrow indicates the magnitude of the standardized coefficient. Correlated errors among “Specific stream power,” “Summer flow,” and “Bank-full flow” as well as between “TN” (total nitrogen) and “Sulfate” were fit in the models but are not depicted in the diagrams for simplicity. Predictor variables are color-coded by driver category. Ag, agricultural disturbance; Non-ag, nonagricultural disturbance; Rp-S, riparian area at the stream site; Rp-W, riparian buffer within the watershed; Ws, watershed.

**FIGURE 5:**
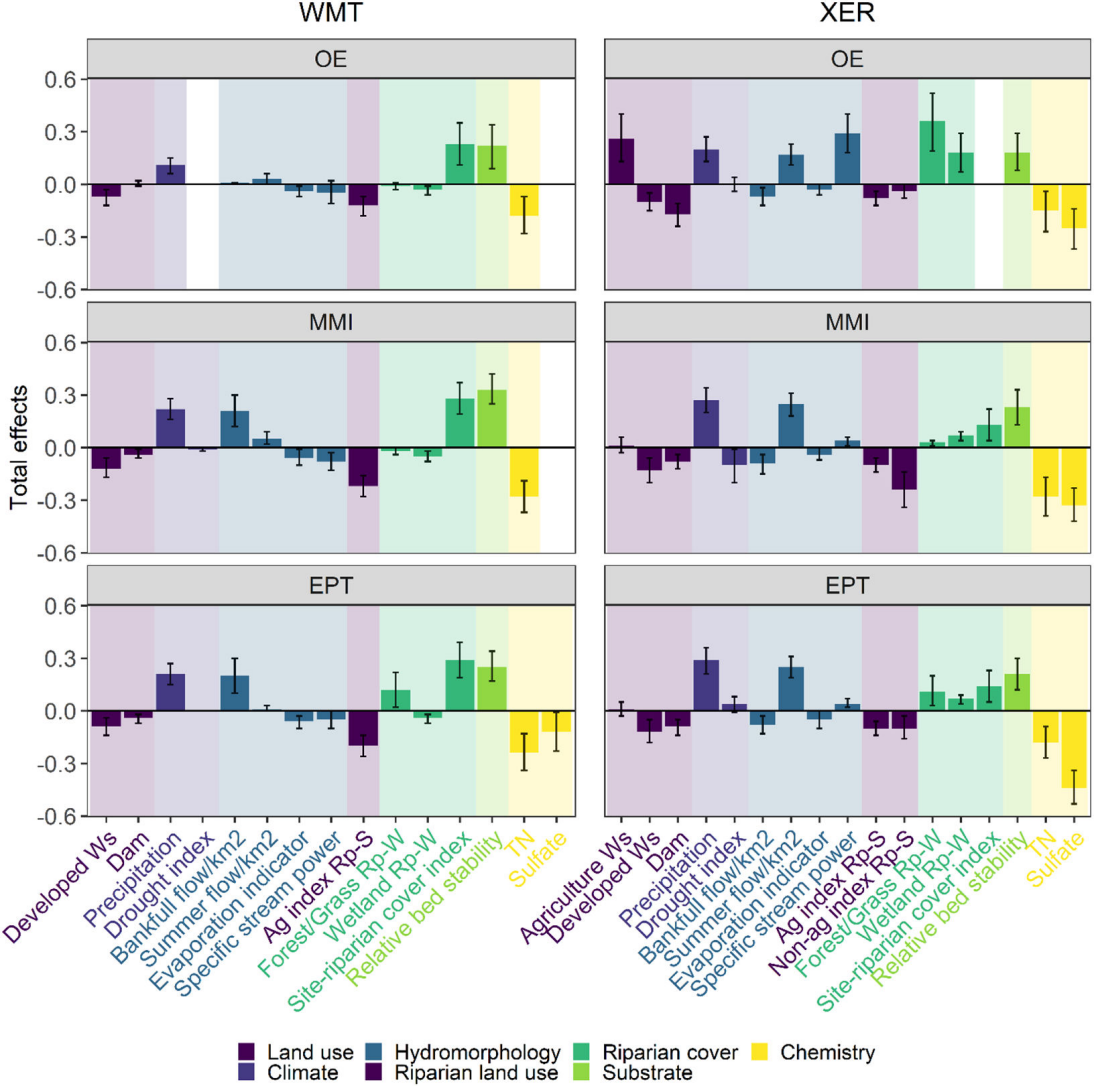
Standardized total effects of predictors on macroinvertebrate biotic indices observed:expected (O/E), multimetric index (MMI), and Ephemeroptera, Plecoptera, Trichoptera taxa richness (EPT) in the Western Mountains (WMT) and Xeric (XER) ecoregions. Predictors are color-coded by driver category. Error bars represent 95% CIs around coefficients. Ag, agricultural disturbance; Non-ag, nonagricultural disturbance; Rp-S, riparian area at the stream site; Rp-W, riparian buffer within the watershed; Ws, watershed.

The main predictors of macroinvertebrate assemblages and their pathways were similar among the three benthic response variables within an ecoregion. But across the two ecoregions, the relative importance of these proximal predictors varied (Figure 4). In the WMT, in-stream physical habitat and water quality variables had the greatest total effects on macroinvertebrate assemblages compared with other predictors (Figure 5). Relative bed stability had direct positive effects and TN had direct negative effects on macroinvertebrate assemblages, as illustrated in the path diagram for MMI and EPT and O/E. Sulfate was negatively associated with EPT richness (Appendix S1: Table S3) but was not related to O/E or MMI in the WMT. In the XER ecoregion, relative bed stability, TN, and sulfate were consistent in-stream drivers of macroinvertebrate assemblages (Figure 5). Sulfate had a greater magnitude of effect on macroinvertebrate assemblages compared with TN and relative bed stability in this ecoregion.

#### Anthropogenic effects

Anthropogenic factors at riparian and watershed scales degraded macroinvertebrate assemblages mostly through indirect effects that operated through in-stream stressors and streamside riparian condition in both ecoregions (Figure 4). Percent developed land in the watershed had negative total effects on O/E, MMI, and EPT in both the WMT and XER ecoregions that were associated with decreased relative bed stability and increased TN concentrations. In the XER ecoregion, developed land degraded macroinvertebrate assemblages through pathways associated with increased bank-full flow, which in turn reduced relative bed stability. Percent agriculture in the watershed was not related to macroinvertebrate assemblages in the WMT nor to MMI and EPT in the XER ecoregion. However, the XER O/E model included a direct positive effect of watershed-level agriculture on O/E, which was unexpected.

In both ecoregions, site-level agricultural disturbance degraded macroinvertebrate assemblages through complex pathways that reduced relative bed stability and riparian cover/complexity and increased nutrient concentrations. In the WMT, site-level agriculture was associated with increased TN through reductions in riparian cover/complexity. In the XER, site-level agriculture effects on macroinvertebrates were partly mediated through reductions in summer flow, which was associated with increased TN and lower bed stability. Site-level nonagricultural disturbance (i.e., urban) degraded macroinvertebrate assemblages in the XER ecoregion (but not WMT) through reduced riparian cover/complexity and increased TN and sulfate concentrations. Dam storage in the watershed negatively affected macroinvertebrates in the XER ecoregion through decreased summer flow, which was related to increased TN and sulfate concentrations, but dam storage had little effect in the WMT.

#### Riparian cover effects

Riparian vegetation cover was positively related to macroinvertebrate assemblages across models, but there were ecoregional differences in the scale and pathways by which these relationships operated. In the WMT, site-riparian cover index affected benthic assemblages indirectly through enhanced relative bed stability and reductions in TN (Figure 4), suggesting that site-level riparian cover may reduce in-stream stressors caused by anthropogenic activities in the WMT. This index also had strong direct effects on O/E (standardized coefficient = 0.16), MMI (0.18), and EPT (0.20) (Appendix S1: Table S3), signifying that woody riparian cover may support macroinvertebrate assemblages through other mechanistic pathways not included in the model (e.g., allochthonous carbon inputs, buffered water temperature). Percent riparian forest/grassland and wetland cover distributed in the watershed were not strongly associated with benthic assemblages in the WMT.

In the XER ecoregion, riparian cover at both the site and watershed scales affected macroinvertebrate assemblages. In contrast to the WMT, the site-riparian cover index was not associated with TN reductions or greater relative bed stability and appeared to affect macroinvertebrates only through direct pathways (Figure 4). Riparian forest/grassland cover and wetlands distributed through the watershed were positively associated with macroinvertebrate assemblages, but the magnitude of their total effects varied. Percent riparian forest/grassland cover had strong direct positive effects on O/E and EPT but was only weakly associated with MMI (Figure 5; Appendix S1: Table S4). Percent riparian wetlands in the XER indirectly affected all three macroinvertebrate assemblage variables through complex pathways associated with increased summer flow, which in turn was related to reductions in TN and sulfate concentrations and greater relative bed stability (Figure 4).

#### Climate and hydrology effects

Macroinvertebrate assemblages in the WMT and XER were affected by annual precipitation through pathways associated with increased relative bed stability and stream flow (bank-full flow in WMT and summer flow in XER). Drought index had little effect on macroinvertebrates across the models except for a negative direct effect on MMI in the XER ecoregion (Figure 4). Stream hydrology effects varied by ecoregion. In the WMT, bank-full flow had positive direct effects on MMI (0.20) and EPT (0.14) but was not related to O/E (0.01) (Appendix S1: Table S3). In the XER, summer flow indirectly affected O/E (0.17), MMI (0.25), and EPT (0.25) through decreased TN and sulfate concentrations and increased relative bed stability (Appendix S1: Table S4). The evaporation indicator was negatively associated with macroinvertebrate assemblages across all models, largely through a positive association with stream chemistry (i.e., increased TN in the WMT and increased sulfate in XER). Specific stream power, an indicator of potential stream hydraulic energy, was positively associated with macroinvertebrates in the XER, but the magnitude of this relationship was greater on O/E (0.29) compared with MMI (0.04) and EPT (0.04).

## DISCUSSION

Our path analysis model of natural and anthropogenic drivers of stream macroinvertebrate assemblages was supported by empirical data from the Western CONUS and aligned with our three hypotheses. We found that the three macroinvertebrate assemblage variables were related to similar predictors within an ecoregion. This demonstrated that O/E, MMI, and EPT richness, although derived from separate methods and characterizing different facets of macroinvertebrate assemblages, captured aspects of biological condition that responded similarly to natural and anthropogenic drivers. These robust relationships in the path analysis models lend empirical support for the hypothesized causal representation of factors affecting stream macroinvertebrates. Comparing path analysis models across ecoregions highlighted how regional attributes like climate and geological characteristics influenced how proximal and watershed factors interacted to affect stream biota. We further discuss the path analysis models’ support of the hypotheses and emphasize the unique insights that an SEM analytic approach can provide. In addition, we elaborate on how the findings can help guide management of western streams.

### Hypothesis 1: Proximal in-stream variables are primary drivers of macroinvertebrate assemblages

Proximal environmental characteristics related to in-stream substrate, physical habitat, and water chemistry had the greatest total effect on macroinvertebrate assemblages in the path analysis models. In general, unstable streambeds with excess fines and sands and elevated nitrogen and sulfate concentrations were associated with degraded macroinvertebrate assemblages. These relationships are widely recognized in stream ecology with multiple studies demonstrating macroinvertebrate indices having positive associations with coarse substrate (Herlihy et al., 2020; Jessup et al., 2014; Munn et al., 2009; Riseng et al., 2011; Wahl et al., 2013) and negative associations with elevated TN (Heatherly et al., 2007; Riseng et al., 2011; Waite, 2014). However, our model results quantified the relative importance of each of these drivers taken together and documented how their effects differed across macroinvertebrate assemblage responses and ecoregional setting.

#### Relative bed stability

Increased fine, unstable streambed sediment (decreased relative bed stability) is widely recognized as a major stressor to macroinvertebrate assemblages and other aquatic organisms (Kaufmann et al., 2009; Kaufmann, Hughes, Paulsen, Peck, Seeliger, Kincaid, et al., 2022; USEPA, 2020b). Relative bed stability was one of the prominent drivers of O/E, MMI, and EPT in our models, particularly in the WMT where the total effects of relative bed stability on macroinvertebrate assemblages were of equal or greater magnitude compared with TN and riparian cover effects. Management activities focused on protecting substrate integrity and reducing excess fines could greatly improve macroinvertebrate assemblage condition. An attributable risk analysis in Washington State indicated that over 50% of stream kilometer length considered to be in poor biotic condition, based on MMI, could be improved by protecting relative bed stability (Larson et al., 2019). In the broader western US region, it was estimated that reductions in excess fine sediment could improve 28% of stream length considered to have poor macroinvertebrate condition (Kaufmann, Hughes, Paulsen, Peck, Seeliger, Kincaid, et al., 2022). More work is needed in developing regional sediment criteria to protect stream biotic assemblages from excess fine sediment (Bryce et al., 2010) and guide management actions to realize potential stream improvements. Our path analysis models highlight the overarching importance of stream substrate condition on macroinvertebrate assemblages after accounting for other potential environmental stressors and point toward the need for integrative management activities that protect streambed condition.

#### Water quality

Elevated nutrients such as TN are recognized stressors on stream biotic condition (e.g., USEPA, 2020b), but these effects can be variable among aquatic assemblages and across landscape contexts. Identifying these different relationships can support better meeting management objectives. We found that the magnitude of TN effects varied among macroinvertebrate response variables, such that within an ecoregion, TN effects were greater on MMI compared with TN effects on O/E or EPT. These differences may be attributed to how the macroinvertebrate response variables were defined and derived (Hawkins, 2006). For example, the multimetric indices included percent tolerant taxa in their composite scores, which are expected to be more responsive to water quality stressors compared with general community richness metrics that may not exhibit uniform responses. The relative effects of TN on macroinvertebrate assemblages were similar across the two ecoregions, indicating that elevated TN is a broadscale stressor to stream biotic condition in the west.

In the XER, sulfate concentrations had greater total effects on degraded macroinvertebrate assemblages than did TN. In the western United States, sulfate concentrations likely originate from natural watershed geology and weathering processes (Mast et al., 2011; Todd et al., 2012) and from land use activities, such as mining, agriculture, and impervious surfaces (Miltner, 2021; Szynkiewicz et al., 2011). While high sulfate concentrations are associated with declines in aquatic insect taxa, such as mayflies (Miltner, 2021), elevated sulfate by itself may not be significantly harmful but rather could be indicative of mining and other human land use activities that can release a mixture of major ions and metals into streams that are toxic to macroinvertebrates (Clements & Kotalik, 2016; Kimmel & Argent, 2019). Mine densities as low as ≤0.01 mines have been associated with threshold responses in fish assemblages and can impair stream biological condition (Daniel et al., 2015). Historically in the Western United States, hard rock mining and rock milling predate regulatory oversight (Woody et al., 2010). These inactive and abandoned mines are widespread, but poorly documented and may pose significant risks to human health and the environment (Durkin & Herrmann, 1994; Sims, 2011). We lacked data on the locations of historic mine sites and mining-associated contaminants to further investigate this hypothesis.

The path analysis models identified possible mechanisms by which sulfate and other covarying contaminants may be transported to streams from landscape disturbances in the arid west. We found higher sulfate concentrations in watersheds with low precipitation, low summer flows, and evaporated stream water. Warmer temperatures and drought conditions have been associated with watershed weathering processes tied to increases in stream sulfate concentrations (Mast et al., 2011; Todd et al., 2012). Also in locations with low annual precipitation, large storm events can mobilize and transport substantial loads of sediment and contaminants to downstream areas (Sims, 2011). Identifying the hydrologic and climatic conditions by which contaminants are transported to streams is critical for pollution management.

### Hypothesis 2: Anthropogenic land use indirectly affects macroinvertebrate assemblages

Land use activities operate at multiple scales and through complex pathways to affect stream ecosystem structure and function (Allan, 2004; Heatherly et al., 2007). Our model supported the hypothesis that anthropogenic land use primarily affected macroinvertebrates indirectly through proximal environmental stressors. These relationships align with other studies that used structural equation models to identify indirect pathways by which agriculture and urban land use affected macroinvertebrate assemblages through impaired physical habitat and degraded water quality (Maloney & Weller, 2011; Riseng et al., 2011; Schmidt et al., 2018).

In our study, urban development in the watershed negatively affected macroinvertebrate assemblages through variable pathways that followed urban stream syndrome effects (Walsh et al., 2005). In the WMT, developed land in the watershed degraded macroinvertebrate assemblages through poor water quality (increased TN) and physical habitat condition (decreased relative bed stability). Developed land use activities promote nutrient and fine sediment transport to streams in this ecoregional setting that under natural conditions would yield relatively low sediment delivery (Booth et al., 2016). In the XER ecoregion, developed land in the watershed impaired macroinvertebrate assemblages through more complex pathways compared with the WMT. Developed land in the XER was associated with degraded water quality (increased TN and sulfate) similar to the WMT, but also was related to increased bank-full flow, which in turn led to reduced relative bed stability. These relationships align with the observations that urban development in the arid west results in flashier hydrographs with large bank-full-flow events that cause bank erosion and channel incision and lead to excessive sediment and fines in the stream (Walsh et al., 2005).

Agricultural activity in the Western United States impacts streams through multiple pathways that include reducing riparian habitat, acting as a nonpoint source of nutrient pollutants, and significantly altering stream hydrologic regimes through water withdrawals and diversions for irrigation, which in turn affect channel morphology and substrate composition (Burdon et al., 2013; Hughes & Vadas, 2021; Riseng et al., 2011). In our study, agriculture had variable effects on macroinvertebrate assemblages that were influenced by the scale of the metric. Streamside agricultural disturbance had significant negative total effects on macroinvertebrate assemblages in the WMT and XER ecoregions that operated through reductions in summer and bank-full flows and relative bed stability. At the watershed scale, agriculture did not significantly affect macroinvertebrate assemblages, except for a direct positive association with O/E in the XER ecoregion. This positive relationship was unexpected and may be due to a correlative effect between agriculture and missing environmental variables not represented in the path model. For example, agriculture might be associated with greater water presence in this arid ecoregion, but we may not have adequately represented this effect in the path model. This spurious result highlights a limitation of SEMs when conceptual models are incomplete or lack representative variables.

In the Western United States, where seasonality affects water presence, dams are widespread and serve multiple purposes (Poff & Hart, 2002). Dams alter stream ecological functions through altered flow regimes, disruption of sediment transport, floodplain disconnection, and fragmentation of river corridors (Poff et al., 2007), all of which affect habitat condition and macroinvertebrate assemblages (Carlisle et al., 2017; Herlihy et al., 2020). Dam effects on macroinvertebrates in the path analysis model were more pronounced in the XER ecoregion, where dam storage in the watershed was associated with reduced summer flows and greater evaporation, which in turn was related to increased TN and sulfate concentrations and reduced relative bed stability. We expected that dams would have more widespread impacts on western streams. But our model may not have revealed these effects because of the coarse nature of the dam storage variable. Future studies may benefit from compiling more spatially explicit dam information to include in the models.

Anthropogenic effects on stream ecosystems and macroinvertebrate assemblages are variable in the Western United States and are likely to be influenced by changing climate and evolving human water needs. To meet these challenges, landscape metrics characterizing anthropogenic infrastructure (e.g., stormwater drainage networks) and land cover conversion may help clarify the impact of anthropogenic activities on stream ecosystems and how these relationships may change in the future.

### Hypothesis 3: Riparian cover reduces land use effects on macroinvertebrate assemblages

In both the WMT and XER ecoregions, site-level riparian cover and structural complexity conveyed direct benefits to macroinvertebrate assemblages that appear to be independent of land use in the model. The specific mechanisms (e.g., thermal buffering, woody debris source) underlying these relationships are unclear because we lacked data or did not have access to potential mediating variables in the model (e.g., water temperature). But the strength of these relationships demonstrated that riparian cover in the Western United States supports stream biotic condition independent of broadscale land use activities.

In the XER ecoregion, the total effects of site-level riparian cover and complexity on macroinvertebrate assemblages were smaller and operated through simpler pathways compared with the WMT. In the arid southwest (XER), riparian vegetation has declined in area and structural complexity due to water resource development, altered flow regimes, floodplain clearing for agriculture and urbanization, and invasion by non-native riparian vegetation species (Ringold et al., 2008; Stromberg et al., 2007; Swift, 1984). Land use activities at multiple scales directly degraded riparian cover in the XER ecoregion. In addition, the model indicated that dam effects indirectly reduced riparian cover and structural complexity through altered stream hydrology (specific stream power).

Riparian buffers have been shown to be effective management tools that provide multiple benefits to streams, but these effects are variable across studies. We found that the ecoregional setting and scale of riparian cover affected these relationships. In the WMT, site-level riparian cover (riparian cover immediately adjacent to the sampling site) was associated with enhanced stream substrate condition and reduced TN concentrations, apparently reducing the adverse influences of developed land use. These effects were prominent: the total positive effects of riparian cover exceeded the total negative effects of urban development in the watershed. Protecting and restoring site-level riparian cover may offer a cost-effective management tool to support stream biotic condition in the WMT.

In contrast to results in the WMT, site-level riparian cover effects on macroinvertebrate variables in the XER ecoregion were modest and apparently overwhelmed by the cumulative negative effects of developed land and dams in the watershed. While site-level riparian cover provided direct benefits to macroinvertebrate assemblages, possibly through thermal buffering or as a source of organic matter, these benefits were not associated with reducing land use-related stressor effects such as improving water quality or substrate condition. Riparian cover distributed throughout the stream watershed, however, was associated with improved in-stream conditions. Riparian wetlands were related to reduced TN and sulfate concentrations that were completely mediated through increased summer flows. These pathways were more closely related to altered hydrologic flows related to dams, not land use, and imply that riparian cover may have limited effects on mitigating urban or agricultural land use impacts where the hydrology of streams is greatly altered by dams and irrigation withdrawal in the XER ecoregion.

Studies show that intensive land use may limit the effectiveness of riparian cover (Wahl et al., 2013; Walsh et al., 2005), and broader watershed-level management plans are needed to support stream ecosystems more effectively. Streams in urban settings may not respond strongly to riparian buffers because of altered landscape flowpaths, for example, impervious surfaces and stormwater drainage systems, that facilitate landscape runoff to bypass riparian vegetation and flow directly into streams (Allan, 2004). Stormwater management that maintains natural streambed disturbance regimes (Hawley et al., 2016) and restores natural fluvial dynamics (Stromberg et al., 2007) may be more effective than efforts simply focused on riparian cover for combating urban-related stressors on streams. In addition, intensive agricultural activities in the watershed can overwhelm riparian benefits when nutrient and pollutant loadings exceed the rates at which riparian vegetation can remove and immobilize nutrients and pollutants (Cole et al., 2020). In these scenarios, riparian buffers are not sufficient to support macroinvertebrate assemblages, and additional measures are needed to reduce nutrient and pollutant loadings from their sources in the watershed (Le Gall et al., 2022). Riparian restoration coupled with other local and watershed-scale management activities are likely to enhance stream ecosystem condition in the XER ecoregion. Using SEM approaches to disentangle the relative effects of riparian cover in relation to land use across different ecoregional settings can be a useful tool to inform management.

### Model limitations and considerations

The path analysis models provide quantitative support for hypothesized pathways by which various natural and anthropogenic factors affect stream macroinvertebrate assemblages. However, these models are an abstraction of complex processes occurring on the landscape and have limitations. An attractive aspect of SEM is the ability to draw causal inference from well-developed theoretical models (Bollen & Pearl, 2013; Grace & Bollen, 2005). However, our model is composed of spatially and temporally coarse predictor variables spanning broad geographic extents, and we take caution in interpreting path coefficients as support for causal effects when it is likely that intermediary factors and processes are missing from the model. Correlative relationships and exclusion of important mediating variables may complicate interpretation and possibly account for observed spurious relationships. However, many of the strong relationships we found in the model results reinforced the underlying theory used to construct the path analysis models and lend validity to the SEM approach. Furthermore, the interactive network of associations we summarize with our SEM results is consistent with that of more narrowly focused studies and with understanding of mechanistic processes, indicating at broad spatial extents, these mechanistic relationships are still applicable to spatial variation in macroinvertebrate assemblages. In this sense, our results contribute to the weight of evidence for causal interpretation of the major associations we have discussed.

The variable explanatory power of our path analysis models was another limitation. The model $R^{2}$ values ranged from 0.14 to 0.43, signifying that over half the variation in macroinvertebrate indices was not accounted for in the models. Possible reasons for the modest model performance may be the exclusion of important predictors in the model, misalignment of spatial and temporal scales between predictors and responses, and variable precision (noise). We could not include all hypothesized predictors of macroinvertebrate assemblages in our model because we lacked data at our study extent. Pesticides and herbicides can significantly impact macroinvertebrate assemblages (Schmidt et al., 2018), but data on these contaminants are not available at a national extent. In addition, our model did not include information on livestock grazing, which is prevalent in the Western US rangelands and associated with degraded stream physical habitat, water quality, and biotic conditions (Hughes & Vadas, 2021). Future analyses should evaluate the long-term and contemporary effects of livestock grazing on aquatic ecosystem structure and function. Water temperature is known to affect macroinvertebrate community composition and secondary production (Munn et al., 2009; Patrick et al., 2019) but was not included in the model. Modeled water temperature in our dataset was used to derive *E* in the O/E metric and was excluded as a predictor in the path analysis model to avoid circularity. A measure of the deviation of stream temperature from reference condition would be a useful predictor for this study to assess changes in climate effects on macroinvertebrates, but is not currently available.

The issue of scale influencing relationships is well recognized in ecology (Levin, 1992) and was a factor in this study as well. The NRSA in situ data are snapshots of stream conditions at the time field crews visited the sites, and these characteristics may not correspond with the landscape and climate predictors in the model that were collected and aggregated over different periods of time. We tried to minimize temporal mismatch in predictor and response variables by matching the year that landscape and climate predictors were collected with the temporal period of the NRSA survey. However, past land use activities can have lasting, legacy effects on stream ecosystems and can be better indicators of taxonomic assemblages than contemporary land use (Harding et al., 1998; Leal et al., 2016; Santos et al., 2020). Incorporating information on land use conversion may be something to consider in future studies to help improve prediction.

Finally, precision in predictor and response variables may have affected model performance, particularly for O/E models. Macroinvertebrate O/E had low precision based on S:N values in both the WMT (1.74) and XER (2.93) ecoregions, indicating that variance of O/E among stream sites was only 1.7 and 2.93 times greater than variance between visits in the WMT and XER ecoregions, respectively. These relatively low S:N values limit the response of O/E to landscape predictors and may account for the low $R^{2}$ in the O/E models. The precision of variables should be taken into account when developing path analysis models and interpreting results.

### Conclusions

Path analysis models provide an accessible quantitative framework to disentangle the relative effects of natural and anthropogenic factors on macroinvertebrate assemblages. The results of our models supported our hypothesized conceptualization of proximal and distal drivers of stream macroinvertebrate assemblages. We found that proximal, in-stream factors had the greatest magnitude of effect on macroinvertebrates, and watershed- and site-level land use effects were mediated through in-stream stressors. More importantly, we identified subtle but important ecoregional differences in predictor–response relationships among the distinct western US subregions. Recognizing these ecoregional differences is critical to developing appropriate and effective management activities. Applying an SEM framework to address complex ecological questions is a powerful approach to explicitly evaluate hypothesized causal pathways by which multiscaled factors shape stream ecosystems. This approach is also well suited to leverage the potential of regional and national ecological datasets to examine broadscale similarities and differences and improve stream assessments.

## Supplementary Material

Supplement1

## Figures and Tables

**TABLE 1:** Description of variables in path analysis model.

Driver category	Variable label	Data source	Description	Transformation
Climate	Precipitation	StreamCat	Total annual precipitation (mm) during the survey water year (May of previous year to April of sampling year).	NT
	Drought index	NOAA	Mean annual drought index during the survey year calculated as −1 × (Palmer Hydrologic Drought Index).	NT
Land use	Agriculture Ws	StreamCat	% upstream watershed area classified as agricultural (row crop + pasture hay).	Arcsine(√(*x*/100))
	Developed Ws	StreamCat	% upstream watershed area classified as developed (open space + low- to high-intensity land use).	Arcsine(√(*x*/100))
	Dam	StreamCat	Ratio of dam storage in the upstream watershed to watershed runoff.	Log_10_(*x* + 0.001)
Hydromorphology	Specific stream power	NRSA	Index of specific stream power calculated as (stream slope × thalweg depth)/mean wetted width.	Log_10_(*x*)
	Bank-full flow	NRSA	Estimated bank-full discharge (m^3^ s^−1^ km^−2^) modeled from stream morphometry attributes and scaled by watershed area.	Log_10_(*x*)
	Summer flow	NRSA	Estimated summer low flow discharge (m^3^ s^−1^ km^−2^) modeled from stream morphometry attributes and scaled by watershed area.	Log_10_(*x*)
	Evaporation indicator	NRSA	Indicator of evaporation where higher values indicate greater evaporation. Calculated from d-excess, an index of evaporative effects on water isotope ratios relative to equilibrium effects.	NT
Riparian	Forest/grass Rp-W	StreamCat	% upstream watershed area within 100 m buffer of stream classified as natural terrestrial cover (deciduous, evergreen, and mixed forest + shrub/scrub + grassland).	Arcsine(√(*x*/100))
	Wetland Rp-W	StreamCat	% upstream watershed area within 100 m buffer of stream classified as wetland (woody + herbaceous).	Arcsine(√(*x*/100))
	Ag index Rp-S	NRSA	Proximity-weighted index of human agricultural disturbance (row crops, pastures) based on field observations of presence and proximity to the stream sampling site.	NT
	Non-ag index Rp-S	NRSA	Proximity-weighted index of human non-agricultural disturbance (buildings, landfill/trash, logging, mining, lawns, pavement, pipes, walls, or revetments) based on field observations of presence and proximity to the stream sampling site.	NT
	Site-riparian cover index	NRSA	A visual index of woody riparian cover and complexity based on field visual estimates of areal cover in three vegetative layers (ground, mid, canopy) within 10 m of the sampling site.	Log_10_(*x* + 0.01)
Substrate	Relative bed stability	NRSA	Relative bed stability index calculated as the ratio of the geometric mean stream particle diameter (mm) to maximum diameter (mm) mobile at bank-full flow.	Log_10_(*x*)
Chemistry	TN	NRSA	Total nitrogen concentration (mg L^−1^)	Log_10_(*x* + 0.01)
	Sulfate	NRSA	Sulfate concentration (mg L^−1^)	Log_10_(*x*)
Macroinvertebrate assemblages	O/E	NRSA	Ratio of observed benthic macroinvertebrate assemblage taxa richness relative to expected least-disturbed (regional reference) benthic richness	NT
	MMI	NRSA	Multimetric index of benthic macroinvertebrate condition composition	NT
	EPT	NRSA	Ephemeroptera, Plecoptera, and Trichoptera richness metric	NT

*Note*: Variables derived from field measurements come from National Rivers and Streams Assessment (NRSA) and variables derived from geographic data layers come from the StreamCat database or National Oceanic and Atmospheric Administration (NOAA).

Abbreviation: NT, not transformed.

**TABLE 2:** Estimated measurement variance (S:N = signal-to-noise) and maximum $R^{2}$ (max $R^{2}$) for response variables in the path analysis models by ecoregion.

	WMT		XER	
Response variable	S:N	Max $R^{2}$	S:N	Max $R^{2}$
O/E	1.74	0.64	2.93	0.74
MMI	4.18	0.81	2.47	0.71
EPT	3.73	0.79	4.05	0.80
Relative bed stability	12.85	0.93	3.70	0.79
Site-riparian cover index	9.00	0.90	17.50	0.94
Summer flow	9.63	0.91	9.87	0.91
Bank-full flow	14.50	0.94	8.49	0.89
Specific stream power	38.90	0.97	6.29	0.86
Evaporation index	1.96	0.66	2.00	0.67
TN	6.25	0.86	7.12	0.88
Sulfate	145.67	0.99	88.81	0.99

Abbreviations: EPT, Ephemeroptera, Plecoptera, Trichoptera taxa richness; MMI, multimetric index; O/E, observed:expected; TN, total nitrogen; WMT, Western Mountains ecoregion; XER, Xeric ecoregion.

**TABLE 3:** Model fit statistics for path analysis models by macroinvertebrate response and ecoregion.

Ecoregion	Response	*χ*^2^ (df)	CFI	TLI	RMSEA	SRMR	*n*
WMT	O/E	63.82 (42)	0.98	0.96	0.04	0.04	320
	MMI	82.08 (48)	0.97	0.96	0.05	0.05	320
	EPT	98.92 (59)	0.97	0.95	0.05	0.05	320
XER	O/E	98.41 (54)	0.97	0.94	0.06	0.04	271
	MMI	107.43 (67)	0.97	0.95	0.05	0.04	272
	EPT	106.66 (68)	0.97	0.96	0.05	0.04	272

Abbreviations: CFI, comparative fit index; EPT, Ephemeroptera, Plecoptera, Trichoptera taxa richness; MMI, multimetric index; *n*, number of observations; O/E, observed:expected; RMSEA, root mean square error of approximation; SRMR, standardized root mean square residual; TLI, Tucker–Lewis index; WMT, Western Mountains ecoregion; XER, Xeric ecoregion.

**TABLE 4:** Coefficients of determination ($R^{2}$) for response (endogenous) variables in path analysis models by macroinvertebrate assemblages and ecoregion.

	WMT			XER		
Response	O/E	MMI	EPT	O/E	MMI	EPT
Macroinvertebrate assemblage	0.14	0.38	0.36	0.32	0.43	0.43
Site-riparian cover index	0.30	0.30	0.30		0.24	0.24
Specific stream power	0.22	0.22	0.22	0.37	0.37	0.37
Summer flow	0.30	0.33	0.33	0.36	0.36	0.36
Bank-full flow	0.37	0.37	0.37	0.35	0.35	0.35
Evaporation indicator	0.33	0.33	0.33	0.47	0.48	0.48
Relative bed stability	0.22	0.22	0.22	0.20	0.21	0.21
TN	0.24	0.24	0.23	0.27	0.27	0.27
Sulfate			0.23	0.39	0.39	0.39

*Note*: Empty cells indicate response variables not included in the final model.

Abbreviations: EPT, Ephemeroptera, Plecoptera, Trichoptera taxa richness; MMI, multimetric index; O/E, observed:expected; TN, total nitrogen; WMT, Western Mountains ecoregion; XER, Xeric ecoregion.

## Data Availability

Data are published and publicly available. Original data come from the US EPA NRSA surveys (https://www.epa.gov/national-aquatic-resource-surveys/data-national-aquatic-resource-surveys) and StreamCat (https://www.epa.gov/national-aquatic-resource-surveys/streamcat-dataset).
