# Supplementary material for: Disentangling natural and anthropogenic effects on benthic macroinvertebrate assemblages in western US streams
Source: Ecosphere. Author manuscript; Available in PMC 2024 Nov 9. (PMC11235210; doi:10.1002/ecs2.4688)
Supplement: Supplement1 [file NIHMS1995635-supplement-Supplement1.docx]

Appendix S1: Supporting information

Table S1. Variables included in the path analysis models of benthic macroinvertebrate assemblages in western wadeable streams, empirical support for their inclusion, and predicted effects on benthic macroinvertebrate condition.

| Driver category | Variable | Support for inclusion in model | Predicted effects |
| --- | --- | --- | --- |
| Land use | Developed Ws | Urban development alters landscape form and watershed hydrology by disturbing soil, removing natural vegetation communities, adding impervious surfaces, and can directly alter channel morphology. Developed land can contribute point and non-point source pollutants, pesticides, herbicides, heavy metals, and fertilizer to receiving waterbodies.    (Heatherly et al. 2007, Helms et al. 2009, Maloney and Weller 2011, Schmidt et al. 2018) | Negative |
|  | Agriculture Ws | Agricultural land is a non-point source of nutrients, herbicides, and pesticides to receiving waterbodies. Tillage and removal of natural vegetation and irrigation can lead to altered streamflow, increased erosion, and impaired instream habitat condition. Agricultural activities can also alter  (Maloney and Weller 2011, Riseng et al. 2011, Schmidt et al. 2018, Meißner et al. 2019) | Negative |
|  | Dam | Dams alter stream flow regimes and reduce longitudinal connectivity and movement of nutrients and sediment from upstream areas of the watershed.  (Carlisle et al. 2017, Hughes and Vadas Jr 2021) | Negative |
| Climate | Precipitation | Precipitation affects watershed runoff and stream hydrologic regimes which in turn affect nutrient and pollutant transport. Stream hydrologic regimes affect instream substrate stability and habitat characteristics.  (Helms et al. 2009, Patrick et al. 2019) | Positive/ negative |
|  | Drought Index | Prolonged dry periods negatively affect aquatic biota especially taxa that lack strategies to survive drying and that are immobile. But the degree of drought impacts on macroinvertebrates are variable and influenced by multiple factors such as the length and severity of the drought, stream hydromorphological characteristics that can provide resilience to drying, recolonization by nearby intact habitat, hysteresis of the climate-groundwater system, and nearby land use activities and water extraction.  (Boulton 2003, Beschta et al. 2013) | Negative |
| Riparian land use | Ag index Rp-S | Agricultural activity in the riparian area of streams are associated with poor streambed stability, lower density of woody riparian vegetation, and poor instream fish habitat.  (Hughes and Vadas Jr 2021, Kaufmann et al. 2022a, Kaufmann et al. 2022b, Palt et al. 2022) | Negative |
|  | Non-ag index Rp-S | Urban developed land near the stream can alter channel morphology and hydrologic dynamics.  (Palt et al. 2022) | Negative |
|  | Forest/Grass Rp-W | Riparian forest is associated with reduction in sediment, nutrients, and pesticides in watersheds with land use activities. Riparian forest can buffer stream water temperature by providing shade. It also is a source of allochthonous organic materials.  (Feld et al. 2018, Schmidt et al. 2018, Hughes and Vadas Jr 2021, Kaufmann et al. 2022b, Palt et al. 2022) | Positive |
|  | Wetland Rp-W | Riparian wetlands can retain nutrients and pesticides from entering the stream. But they also enhance lateral connectivity that connects the stream to the floodplain.  (Riseng et al. 2011, Feld et al. 2018) | Positive |
| Hydromorphology | Specific stream power | An index of specific stream power reflecting the force of moving water on the streambed that affects channel scouring, hydraulic retention, and the size and stability of streambed particles that comprise macroinvertebrate habitat. The index is derived from channel morphology attributes.  We expect that specific stream power is affected by climate (e.g., precipitation), geomorphology, channel gradient, nearshore land cover, land use, and water extraction.  (Kaufmann personal communication) | Positive/ negative |
|  | Summer flow/km2 | Summer low flow determines the amount of water in a channel, and is typically the limitation on habitat space for aquatic biota. In addition, streams with summer flows that are large enough to bring woody debris and coarse particulate organic matter to enter from the nearshore landscape and deep enough to buffer from warming temperature may support more diverse and intact macroinvertebrate assemblages.  We expect that summer flow is affected by climate (e.g., precipitation), geomorphology, nearshore land cover, land use, and water extraction activities.  (Kaufmann et al. 2008, Feld et al. 2018, Meißner et al. 2019, Patrick et al. 2019) | Positive |
|  | Bankfull flow/km2 | High bankfull flows are associated with impaired macroinvertebrate assemblages that may be related to flashier flows that scour and erode stream beds, impair substrate and habitat condition, displace organisms, and alter thermal and decomposition dynamics.  We expect that bankfull flow is affected by climate (e.g., precipitation), geomorphology, nearshore land cover, land use, and water extraction activities.  (Kaufmann et al. 2008, Helms et al. 2009, Carlisle et al. 2017, Patrick et al. 2019) | Negative |
|  | Evaporation indicator | Indicator of the degree of evaporation experienced in the stream water and is related to water retention time in open water pools on the landscape. Associated with increased nitrogen concentrations.  We expect that stream water evaporation is affected by climate, stream hydromorphology, land cover, land use, and water regulation activities.  (Brooks et al. 2014) | Negative |
| Nearshore cover | Site-riparian cover index | Intact riparian cover can slow runoff, retain sediments, nutrients, and other pollutant inputs to streams, promote bank stabilization, provide organic inputs to streams, limit in-stream primary productivity through canopy shading, and reduces stream temperature (thermal damping).  We expect that woody riparian cover and structural complexity is affected by climate, land cover in the broader watershed, stream hydromorphology, and land use activities.  (Feld et al. 2018, USEPA 2020) | Positive |
| Instream habitat | Relative bed stability | Streams with relatively stable beds generally lack excess amounts of fine sediment that are readily mobilized during typical flood conditions and are likely to support more diverse, intact macroinvertebrate assemblages compared to streams with instable beds and excess fine sediment that impair benthic habitat by filling interstitial spaces between coarse bed materials.  We expect that relative bed stability is affected by geoclimatic and anthropogenic variables that influence upland erosion and instream sediment transport (e.g., geology, soils, land use, precipitation, runoff, channel morphology, slope, specific stream power, and riparian vegetation).  (Kaufmann 1999, Kaufmann et al. 2008, Munn et al. 2009, Larson et al. 2019, Herlihy et al. 2020, USEPA 2020) | Positive |
| Water quality | Nutrients | Elevated total nitrogen is associated with poor stream ecological condition and N loading from the landscape promotes eutrophication and anoxic conditions.  We expect that nutrient concentrations are affected by land use activities, which can be a non-point source of nutrients, stream hydromorphological characteristics that can affect biogeochemical processing, and riparian land cover, which may take up and retain excess nutrients from entering streams.  (Larson et al. 2019, Herlihy et al. 2020, USEPA 2020) | Negative |
|  | Ions | Sulfate is indicative of land use activities that can impair macroinvertebrate assemblages. Sulfate is a common constituent in agricultural fertilizers and associated with mining effluent that may contain ions and metals that are toxic to aquatic organisms.  We expect that ion concentrations are affected by geologic weathering, land use activities disturbing soils, and stream hydromorphology, which may have a diluting effect on ion concentrations.  (Szynkiewicz et al. 2011, Kimmel and Argent 2019, USEPA 2020) | Negative |
|  | Turbidity | Turbidity caused by suspended sediment negatively affects macroinvertebrates by reducing physiological function and degrading habitat condition.  We expect that turbidity is driven by geologic weathering, land use activities, and stream hydromorphological characteristics that can promote the suspension of sediments into the water column.  (Henley et al. 2000) | Negative |

Table S2. Macroinvertebrate community metrics used in calculating the NRSA MMI in the Western Mountains (WMT) and Xeric (XER) ecoregions.

| WMT | XER |
| --- | --- |
| EPT % taxa richness | Non-insect % individuals |
| % individuals in top 5 taxa | % individuals in top 5 taxa |
| Scraper taxa richness | Scraper taxa richness |
| Clinger % taxa richness | Clinger % taxa richness |
| EPT taxa richness | EPT taxa richness |
| Tolerant % taxa richness | Tolerant % taxa richness |

Table S3. Standardized effects (direct, indirect, and total) for predictors of macroinvertebrate condition by biotic response metric in the Western Mountains.

| Response | Predictor | Direct | Indirect | Total |
| --- | --- | --- | --- | --- |
| O/E | Developed Ws |  | -0.07 (-0.12, -0.03) | -0.07 (-0.12, -0.03) |
|  | Dam |  | 0 (-0.01, 0.02) | 0 (-0.01, 0.02) |
|  | Precipitation |  | 0.11 (0.06, 0.15) | 0.11 (0.06, 0.15) |
|  | Specific stream power |  | -0.05 (-0.11, 0.02) | -0.05 (-0.11, 0.02) |
|  | Ag index Rp-S |  | -0.12 (-0.18, -0.06) | -0.12 (-0.18, -0.06) |
|  | Forest/Grass Rp-W |  | -0.01 (-0.03, 0.01) | -0.01 (-0.03, 0.01) |
|  | Wetland Rp-W |  | -0.03 (-0.06, -0.01) | -0.03 (-0.06, -0.01) |
|  | Summer flow/km2 |  | 0.03 (0.01, 0.06) | 0.03 (0.01, 0.06) |
|  | Bankfull flow/km2 |  | 0.01 (0, 0.01) | 0.01 (0, 0.01) |
|  | Evaporation indicator |  | -0.04 (-0.06, -0.01) | -0.04 (-0.06, -0.01) |
|  | Site-riparian cover index | 0.16 (0.04, 0.29) | 0.06 (0.03, 0.1) | 0.23 (0.1, 0.35) |
|  | Relative bed stability | 0.22 (0.1, 0.34) |  | 0.22 (0.1, 0.34) |
|  | TN | -0.18 (-0.28, -0.07) |  | -0.18 (-0.28, -0.07) |
| MMI | Developed Ws |  | -0.12 (-0.17, -0.06) | -0.12 (-0.17, -0.06) |
|  | Dam |  | -0.04 (-0.06, -0.01) | -0.04 (-0.06, -0.01) |
|  | Precipitation |  | 0.22 (0.16, 0.28) | 0.22 (0.16, 0.28) |
|  | Drought index |  | -0.01 (-0.02, 0) | -0.01 (-0.02, 0) |
|  | Specific stream power |  | -0.08 (-0.14, -0.03) | -0.08 (-0.14, -0.03) |
|  | Ag index Rp-S |  | -0.22 (-0.28, -0.16) | -0.22 (-0.28, -0.16) |
|  | Forest/Grass Rp-W |  | -0.02 (-0.04, 0) | -0.02 (-0.04, 0) |
|  | Wetland Rp-W |  | -0.05 (-0.08, -0.02) | -0.05 (-0.08, -0.02) |
|  | Summer flow/km2 |  | 0.05 (0.02, 0.09) | 0.05 (0.02, 0.09) |
|  | Bankfull flow/km2 | 0.20 (0.1, 0.29) | 0.01 (0, 0.02) | 0.21 (0.11, 0.3) |
|  | Evaporation indicator |  | -0.06 (-0.1, -0.01) | -0.06 (-0.1, -0.01) |
|  | Site-riparian cover index | 0.18 (0.09, 0.28) | 0.10 (0.05, 0.14) | 0.28 (0.19, 0.38) |
|  | Relative bed stability | 0.33 (0.24, 0.42) |  | 0.33 (0.24, 0.42) |
|  | TN | -0.28 (-0.37, -0.19) |  | -0.28 (-0.37, -0.19) |
| EPT | Developed Ws |  | -0.09 (-0.14, -0.04) | -0.09 (-0.14, -0.04) |
|  | Dam |  | -0.04 (-0.07, -0.02) | -0.04 (-0.07, -0.02) |
|  | Precipitation |  | 0.21 (0.15, 0.27) | 0.21 (0.15, 0.27) |
|  | Drought index |  | 0 (0, 0) | 0 (0, 0) |
|  | Specific stream power |  | -0.05 (-0.1, 0) | -0.05 (-0.1, 0) |
|  | Ag index Rp-S |  | -0.20 (-0.26, -0.14) | -0.20 (-0.26, -0.14) |
|  | Forest/Grass Rp-W | 0.13 (0.03, 0.23) | -0.01 (-0.02, 0.01) | 0.12 (0.02, 0.23) |
|  | Wetland Rp-W |  | -0.04 (-0.07, -0.02) | -0.04 (-0.07, -0.02) |
|  | Summer flow/km2 |  | 0.01 (0, 0.03) | 0.01 (0, 0.03) |
|  | Bankfull flow/km2 | 0.14 (0.03, 0.25) | 0.06 (0.01, 0.1) | 0.20 (0.1, 0.3) |
|  | Evaporation indicator |  | -0.06 (-0.1, -0.03) | -0.06 (-0.1, -0.03) |
|  | Site-riparian cover index | 0.20 (0.1, 0.31) | 0.09 (0.05, 0.13) | 0.29 (0.19, 0.39) |
|  | Relative bed stability | 0.25 (0.17, 0.34) |  | 0.25 (0.17, 0.34) |
|  | TN | -0.24 (-0.34, -0.14) |  | -0.24 (-0.34, -0.14) |
|  | Sulfate | -0.12 (-0.23, -0.01) |  | -0.12 (-0.23, -0.01) |

Note: Values are standardized path coefficients (Upper, Lower 95% confidence intervals).

Table S4. Standardized effects (direct, indirect, and total) for predictors of macroinvertebrate condition by biotic response metric in the Xeric ecoregion.

| Response | Predictor | Direct | Indirect | Total |
| --- | --- | --- | --- | --- |
| OE | Developed Ws |  | -0.10 (-0.15, -0.05) | -0.10 (-0.15, -0.05) |
|  | Agriculture Ws | 0.23 (0.1, 0.37) | 0.03 (0.01, 0.05) | 0.26 (0.13, 0.39) |
|  | Dam |  | -0.17 (-0.23, -0.11) | -0.17 (-0.23, -0.11) |
|  | Precipitation |  | 0.20 (0.13, 0.27) | 0.20 (0.13, 0.27) |
|  | Drought index |  | 0 (-0.04, 0.03) | 0 (-0.04, 0.03) |
|  | Specific stream power | 0.29 (0.18, 0.39) | 0 (0, 0.01) | 0.29 (0.19, 0.39) |
|  | Ag index Rp-S |  | -0.08 (-0.12, -0.04) | -0.08 (-0.12, -0.04) |
|  | Non-ag index Rp-S |  | -0.04 (-0.08, 0) | -0.04 (-0.08, 0) |
|  | Forest/Grass Rp-W | 0.25 (0.08, 0.43) | 0.10 (0.06, 0.14) | 0.36 (0.19, 0.52) |
|  | Wetland Rp-W | 0.14 (0.02, 0.25) | 0.04 (0.02, 0.07) | 0.18 (0.07, 0.3) |
|  | Summer flow/km2 |  | 0.17 (0.11, 0.23) | 0.17 (0.11, 0.23) |
|  | Bankfull flow/km2 |  | -0.07 (-0.12, -0.02) | -0.07 (-0.12, -0.02) |
|  | Evaporation indicator |  | -0.03 (-0.06, 0) | -0.03 (-0.06, 0) |
|  | Relative bed stability | 0.18 (0.08, 0.29) |  | 0.18 (0.08, 0.29) |
|  | TN | -0.15 (-0.27, -0.03) |  | -0.15 (-0.27, -0.03) |
|  | Sulfate | -0.25 (-0.36, -0.14) |  | -0.25 (-0.36, -0.14) |
| MMI | Developed Ws |  | -0.13 (-0.2, -0.06) | -0.13 (-0.2, -0.06) |
|  | Agriculture Ws |  | 0.01 (-0.03, 0.06) | 0.01 (-0.03, 0.06) |
|  | Dam |  | -0.08 (-0.12, -0.04) | -0.08 (-0.12, -0.04) |
|  | Precipitation |  | 0.27 (0.19, 0.34) | 0.27 (0.19, 0.34) |
|  | Drought index | -0.13 (-0.22, -0.04) | 0.03 (-0.01, 0.06) | -0.10 (-0.2, -0.01) |
|  | Specific stream power |  | 0.04 (0.01, 0.06) | 0.04 (0.01, 0.06) |
|  | Ag index Rp-S |  | -0.10 (-0.14, -0.06) | -0.10 (-0.14, -0.06) |
|  | Non-ag index Rp-S | -0.15 (-0.24, -0.06) | -0.09 (-0.15, -0.03) | -0.24 (-0.34, -0.14) |
|  | Forest/Grass Rp-W |  | 0.03 (0.01, 0.04) | 0.03 (0.01, 0.04) |
|  | Wetland Rp-W |  | 0.07 (0.04, 0.09) | 0.07 (0.04, 0.09) |
|  | Summer flow/km2 |  | 0.25 (0.18, 0.31) | 0.25 (0.18, 0.31) |
|  | Bankfull flow/km2 |  | -0.09 (-0.15, -0.04) | -0.09 (-0.15, -0.04) |
|  | Evaporation indicator |  | -0.04 (-0.07, 0) | -0.04 (-0.07, 0) |
|  | Site-riparian cover index | 0.13 (0.04, 0.22) |  | 0.13 (0.04, 0.22) |
|  | Relative bed stability | 0.23 (0.13, 0.33) |  | 0.23 (0.13, 0.33) |
|  | TN | -0.28 (-0.39, -0.16) |  | -0.28 (-0.39, -0.16) |
|  | Sulfate | -0.33 (-0.43, -0.22) |  | -0.33 (-0.43, -0.22) |
| EPT | Developed Ws |  | -0.12 (-0.18, -0.05) | -0.12 (-0.18, -0.05) |
|  | Agriculture Ws |  | 0.01 (-0.03, 0.05) | 0.01 (-0.03, 0.05) |
|  | Dam |  | -0.09 (-0.14, -0.05) | -0.09 (-0.14, -0.05) |
|  | Precipitation |  | 0.29 (0.21, 0.36) | 0.29 (0.21, 0.36) |
|  | Drought index |  | 0.04 (-0.01, 0.08) | 0.04 (-0.01, 0.08) |
|  | Specific stream power |  | 0.04 (0.02, 0.07) | 0.04 (0.02, 0.07) |
|  | Ag index Rp-S |  | -0.10 (-0.14, -0.06) | -0.10 (-0.14, -0.06) |
|  | Non-ag index Rp-S |  | -0.10 (-0.16, -0.03) | -0.10 (-0.16, -0.03) |
|  | Forest/Grass Rp-W | 0.09 (0.01, 0.18) | 0.02 (0.01, 0.04) | 0.11 (0.03, 0.2) |
|  | Wetland Rp-W |  | 0.07 (0.04, 0.09) | 0.07 (0.04, 0.09) |
|  | Summer flow/km2 |  | 0.25 (0.19, 0.32) | 0.25 (0.19, 0.32) |
|  | Bankfull flow/km2 |  | -0.08 (-0.13, -0.03) | -0.08 (-0.13, -0.03) |
|  | Evaporation indicator |  | -0.05 (-0.1, 0) | -0.05 (-0.1, 0) |
|  | Site-riparian cover index | 0.14 (0.06, 0.23) |  | 0.14 (0.06, 0.23) |
|  | Relative bed stability | 0.21 (0.12, 0.3) |  | 0.21 (0.12, 0.3) |
|  | TN | -0.18 (-0.28, -0.08) |  | -0.18 (-0.28, -0.08) |
|  | Sulfate | -0.44 (-0.53, -0.34) |  | -0.44 (-0.53, -0.34) |

Note: Values are standardized path coefficients (Upper, Lower 95% confidence intervals).

REFERENCES

Beschta, R. L., D. L. Donahue, D. A. DellaSala, J. J. Rhodes, J. R. Karr, M. H. O’Brien, T. L. Fleischner, and C. Deacon Williams. 2013. Adapting to climate change on western public lands: addressing the ecological effects of domestic, wild, and feral ungulates. Environmental management **51**:474-491.

Boulton, A. J. 2003. Parallels and contrasts in the effects of drought on stream macroinvertebrate assemblages. Freshwater Biology **48**:1173-1185.

Brooks, J. R., J. J. Gibson, S. J. Birks, M. H. Weber, K. D. Rodecap, and J. L. Stoddard. 2014. Stable isotope estimates of evaporation: inflow and water residence time for lakes across the United States as a tool for national lake water quality assessments. Limnology and Oceanography **59**:2150-2165.

Carlisle, D. M., T. E. Grantham, K. Eng, and D. M. Wolock. 2017. Biological relevance of streamflow metrics: Regional and national perspectives. Freshwater Science **36**:927-940.

Feld, C. K., M. R. Fernandes, M. T. Ferreira, D. Hering, S. J. Ormerod, M. Venohr, and C. Gutiérrez-Cánovas. 2018. Evaluating riparian solutions to multiple stressor problems in river ecosystems—a conceptual study. Water research **139**:381-394.

Heatherly, T., M. R. Whiles, T. V. Royer, and M. B. David. 2007. Relationships between water quality, habitat quality, and macroinvertebrate assemblages in Illinois streams. Journal of environmental quality **36**:1653-1660.

Helms, B. S., J. E. Schoonover, and J. W. Feminella. 2009. Seasonal variability of landuse impacts on macroinvertebrate assemblages in streams of western Georgia, USA. Journal of the North American Benthological Society **28**:991-1006.

Henley, W., M. Patterson, R. Neves, and A. D. Lemly. 2000. Effects of sedimentation and turbidity on lotic food webs: a concise review for natural resource managers. Reviews in Fisheries Science **8**:125-139.

Herlihy, A. T., J. C. Sifneos, R. M. Hughes, D. V. Peck, and R. M. Mitchell. 2020. The relation of lotic fish and benthic macroinvertebrate condition indices to environmental factors across the conterminous USA. Ecological Indicators **112**:105958.

Hughes, R. M., and R. L. Vadas Jr. 2021. Agricultural effects on streams and rivers: a western USA focus. Water **13**:1901.

Kaufmann, P. R., J. M. Faustini, D. P. Larsen, and M. A. Shirazi. 2008. A roughness-corrected index of relative bed stability for regional stream surveys. Geomorphology **99**:150-170.

Kaufmann, P. R., R. M. Hughes, S. G. Paulsen, D. V. Peck, C. W. Seeliger, T. Kincaid, and R. M. Mitchell. 2022a. Physical habitat in conterminous US streams and rivers, Part 2: A quantitative assessment of habitat condition. Ecological Indicators **141**:109047.

Kaufmann, P. R., R. M. Hughes, S. G. Paulsen, D. V. Peck, C. W. Seeliger, M. H. Weber, and R. M. Mitchell. 2022b. Physical habitat in conterminous US streams and rivers, Part 1: Geoclimatic controls and anthropogenic alteration. Ecological Indicators **141**:109046.

Kaufmann, P. R., P. Levine, E. G. Robison, C. Seeliger, D. V. Peck. 1999. Quantifying Physical Habitat in Wadeable Streams. U.S. Environmental Protection Agency, Washington, D.C.

Kimmel, W. G., and D. G. Argent. 2019. Impacts of point-source Net Alkaline Mine Drainage (NAMD) on stream macroinvertebrate communities. Journal of environmental management **250**:109484.

Larson, C. A., G. Merritt, J. Janisch, J. Lemmon, M. Rosewood-Thurman, B. Engeness, S. Polkowske, and G. Onwumere. 2019. The first statewide stream macroinvertebrate bioassessment in Washington State with a relative risk and attributable risk analysis for multiple stressors. Ecological Indicators **102**:175-185.

Maloney, K. O., and D. E. Weller. 2011. Anthropogenic disturbance and streams: Land use and land‐use change affect stream ecosystems via multiple pathways. Freshwater Biology **56**:611-626.

Meißner, T., B. Sures, and C. Feld. 2019. Multiple stressors and the role of hydrology on benthic invertebrates in mountainous streams. Science of The Total Environment **663**:841-851.

Munn, M. D., I. R. Waite, D. P. Larsen, and A. T. Herlihy. 2009. The relative influence of geographic location and reach-scale habitat on benthic invertebrate assemblages in six ecoregions. Environmental monitoring and assessment **154**:1-14.

Palt, M., M. Le Gall, J. Piffady, D. Hering, and J. Kail. 2022. A metric-based analysis on the effects of riparian and catchment landuse on macroinvertebrates. Science of The Total Environment **816**:151590.

Patrick, C. J., D. McGarvey, J. H. Larson, W. Cross, D. Allen, A. Benke, T. Brey, A. Huryn, J. Jones, and C. Murphy. 2019. Precipitation and temperature drive continental-scale patterns in stream invertebrate production. Science advances **5**:eaav2348.

Riseng, C., M. Wiley, R. W. Black, and M. Munn. 2011. Impacts of agricultural land use on biological integrity: a causal analysis. Ecological Applications **21**:3128-3146.

Schmidt, T. S., P. C. Van Metre, and D. M. Carlisle. 2018. Linking the agricultural landscape of the Midwest to stream health with structural equation modeling. Environmental science & technology **53**:452-462.

Szynkiewicz, A., J. C. Witcher, M. Modelska, D. M. Borrok, and L. M. Pratt. 2011. Anthropogenic sulfate loads in the Rio Grande, New Mexico (USA). Chemical Geology **283**:194-209.

USEPA. 2020. National Rivers and Streams Assessment 2013-2014 Technical Support Document. U.S. Environmental Protection Agency, Office of Water and Office of Research and Development, Washington, D.C.
